# TAL effectors and activation of predicted host targets distinguish Asian from African strains of the rice pathogen *Xanthomonas oryzae* pv. oryzicola while strict conservation suggests universal importance of five TAL effectors

**DOI:** 10.3389/fpls.2015.00536

**Published:** 2015-07-21

**Authors:** Katherine E. Wilkins, Nicholas J. Booher, Li Wang, Adam J. Bogdanove

**Affiliations:** ^1^Plant Pathology and Plant-Microbe Biology Section, School of Integrative Plant Science, Cornell UniversityIthaca, NY, USA; ^2^Graduate Field of Computational Biology, Cornell UniversityIthaca, NY, USA

**Keywords:** transcription activator-like (TAL) effector, single molecule real-time (SMRT) sequencing, RNA-Seq, horizontal gene transfer, plant disease resistance, susceptibility (*S*) genes, population genomics

## Abstract

*Xanthomonas oryzae* pv. oryzicola (Xoc) causes the increasingly important disease bacterial leaf streak of rice (BLS) in part by type III delivery of repeat-rich transcription activator-like (TAL) effectors to upregulate host susceptibility genes. By pathogen whole genome, single molecule, real-time sequencing and host RNA sequencing, we compared TAL effector content and rice transcriptional responses across 10 geographically diverse Xoc strains. TAL effector content is surprisingly conserved overall, yet distinguishes Asian from African isolates. Five TAL effectors are conserved across all strains. In a prior laboratory assay in rice cv. Nipponbare, only two contributed to virulence in strain BLS256 but the strict conservation indicates all five may be important, in different rice genotypes or in the field. Concatenated and aligned, TAL effector content across strains largely reflects relationships based on housekeeping genes, suggesting predominantly vertical transmission. Rice transcriptional responses did not reflect these relationships, and on average, only 28% of genes upregulated and 22% of genes downregulated by a strain are up- and down- regulated (respectively) by all strains. However, when only known TAL effector targets were considered, the relationships resembled those of the TAL effectors. Toward identifying new targets, we used the TAL effector-DNA recognition code to predict effector binding elements in promoters of genes upregulated by each strain, but found that for every strain, all upregulated genes had at least one. Filtering with a classifier we developed previously decreases the number of predicted binding elements across the genome, suggesting that it may reduce false positives among upregulated genes. Applying this filter and eliminating genes for which upregulation did not strictly correlate with presence of the corresponding TAL effector, we generated testable numbers of candidate targets for four of the five strictly conserved TAL effectors.

## Introduction

Plant pathogenic *Xanthomonas* spp. inject transcription activator-like (TAL) effectors into host cells, where these proteins activate genes by binding at promoters (Kay et al., 2007; Römer et al., 2010). A TAL effector may activate a susceptibility (*S*) gene that contributes to disease or may activate a resistance (*R*) gene that results in host defense (Boch et al., 2014). In some cases, TAL effectors also or instead activate genes that appear to be inconsequential, “collateral” targets (Li et al., 2012b; Cernadas et al., 2014; Hu et al., 2014). TAL effector targeting is mediated by a modular central repeat region (CRR), with each repeat recognizing one nucleotide according to a degenerate code resulting in specific binding to a contiguous DNA sequence called the effector-binding element (EBE) (Boch et al., 2009; Moscou and Bogdanove, 2009). Nucleotide specificity of each repeat is determined by the hypervariable positions 12 and 13, together known as the repeat variable diresidue (RVD) (Boch et al., 2009; Moscou and Bogdanove, 2009). Crystal structures showed that only the thirteenth residue interacts with the nucleotide, while the twelfth stabilizes the loop that projects the thirteenth residue into the major groove (Deng et al., 2012; Mak et al., 2012). And, while some amino acids at the twelfth position abolish or dramatically reduce binding affinity, RVDs that share the same thirteenth residue typically have similar binding specificities (Boch et al., 2009; Moscou and Bogdanove, 2009; Yang et al., 2014). The thirteenth residue has therefore been referred to as the base-specifying residue (BSR) (De Lange et al., 2014).

Single TAL effectors often determine the outcome of the host-pathogen interaction (Boch et al., 2014). This is clear for TAL effectors that act as avirulence factors by activating a corresponding *R* gene that blocks disease progression (Gu et al., 2005; Römer et al., 2007; Tian et al., 2014), but it is also true for any TAL effector that upregulates an *S* gene that plays a critical role in disease. An example is TAL effector PthXo1 of the bacterial blight of rice pathogen *X. oryzae* pv. oryzae (Xoo) strain PXO99^A^, which upregulates the *S* gene *OsSWEET11* (also Os8N3; Yang et al., 2006), a member of a large family of paralogous sugar transporters. *OsSWEET11* alleles that lack the PthXo1 EBE confer (genetically recessive) resistance (Chu et al., 2006; Yang et al., 2006; Yuan et al., 2009). This resistance can be overcome by strains harboring distinct TAL effectors however (Antony et al., 2010). Indeed, five distinct TAL effectors from different Xoo strains each activate one of five phylogenetically close members of the *SWEET* gene family that function interchangeably as *S* genes (Yang et al., 2006; Antony et al., 2010; Römer et al., 2010; Yu et al., 2011; Streubel et al., 2013; Richter et al., 2014). Similarly, in citrus canker caused by *X. axonopodis* pv. citri (divided into three types) and *X. axonopodis* pv. aurantifolii (two types), representative strains of all five types contain one unique TAL effector each that activates the *S* gene *CsLOB1*, which is both necessary and sufficient for the formation of the pustules that are a hallmark of this disease (Hu et al., 2014; Pereira et al., 2014).

In light of their determinative nature, knowledge of TAL effectors and their targets enables innovative strategies for disease control. Removal of EBEs from *OsSWEET* gene promoters by genome editing resulted in resistance to a diverse collection of Xoo strains (Li et al., 2012a). Every discovery of a novel *S* gene creates new opportunities to engineer host plants with resistance in a similar way. TAL effector-targeted *R* genes can be exploited as well. By engineering an *R* gene promoter to include EBEs for multiple additional TAL effectors, it is possible to trap a broad spectrum of strains and even different pathogens. This strategy applied to the *Xa27* rice bacterial blight *R* gene yielded plants resistant to strains of Xoo lacking AvrXa27 and to each of 10 tested strains of *X. oryzae* pv. oryzicola (Xoc), which causes the distinct disease bacterial leaf streak of rice (Hummel et al., 2012). More recently, the strategy was shown to be effective with another bacterial blight *R* gene, *Xa10* (Zeng et al., 2015). Broad effectiveness and durability of these *S* and *R* gene-centered strategies depends, however, on knowledge of TAL effector content within and across populations of the pathogen.

Bacterial blight and bacterial leaf streak of rice constrain production of this staple crop in many parts of the world (Nino-Liu et al., 2006). Four Xoo strains have been fully sequenced, KACC10331, MAFF311018, PXO99^A^, and PXO86. These harbor 11, 16, 18, and 18 TAL effectors, respectively. Five bacterial blight *S* genes targeted by eight TAL effectors and three bacterial blight *R* genes each targeted by a unique TAL effector have been identified (reviewed in Boch et al., 2014). By comparison, Xoc is less well-characterized, with only two fully sequenced strains, BLS256 and CFBP7342, which secrete 28 and 24 TAL effectors, respectively (Bogdanove et al., 2011; Booher et al., unpublished), only one identified TAL effector-*S* gene pair (Tal2g of BLS256 and the rice sulfate transporter gene *OsSULTR3;6*), and no known TAL effector-activated *R* genes (Cernadas et al., 2014). In a growth chamber assay of TAL effector gene knockout mutants of BLS256, only two showed consistently reduced virulence (including the *tal2g* knockout) (Cernadas et al., 2014), but it is unclear how sensitive this assay is to differences that might be observed in the field. Furthermore, the assay was carried out on only a single rice genotype (cv. Nipponbare) and might have missed other host genotype-specific virulence contributions of TAL effectors. Generating a comprehensive TAL effector gene knockout library is technically demanding and time consuming, and CFBP7342 TAL effector knockouts have not yet been made and tested.

Sequencing of multiple, diverse Xoc strains is a useful alternative approach to identifying uniquely and broadly important TAL effectors, i.e., those that are highly conserved. TAL effectors that perform redundant functions in disease, or no function at all other than perhaps as material for rapid evolution of new TAL effectors via recombination, are likely to be less conserved. In addition to highlighting TAL effectors most likely to function in virulence, TAL effector conservation would inform the design of *R* gene traps, since the inclusion of EBEs bound by highly conserved TAL effectors would mediate broad resistance. In conjunction with transcript profiling of rice inoculated with each of multiple strains, TAL effector sequences are an enabling resource for identifying candidate targets, using the TAL effector DNA binding code for EBE prediction (e.g., Doyle et al., 2012). In particular, TAL effector targets that are activated by many strains even in the absence of TAL effector conservation could represent *S* genes to which convergent evolution has resulted in multiple corresponding TAL effectors, or conversely, to which diversifying selection by an *R* gene has resulted in several related but distinct corresponding TAL effectors.

Here, we report comparative analyses of whole genome sequences of 10 Xoc strains from diverse locations (including BLS256, CFBP7342, and eight more sequenced for this study) and of rice transcriptional responses to each strain, with a focus on TAL effectors and their known and candidate targets. Our results allow inference regarding the importance of particular TAL effectors and targets prior to experimentation, and the potential of TAL-effector-centered strategies for durable resistance to bacterial leaf streak.

## Materials and methods

### Xoc strains and genome sequences

Xoc strains used in this study are given in Table 1. Genome sequences and raw sequence data are available under NCBI BioProject numbers PRJNA280380 and PRJNA283315, respectively. Raw data in.bas.h5/.bax.h5 format are available on request.

**Table 1:** **Xoc strains used in this study**.

**Strain**	**Country of isolation**	**Year of isolation**	**Source or references**
BLS256	Philippines	1984	Vera Cruz^a^
BLS279	Philippines	1984	Vera Cruz^a^
CFBP2286	Malaysia	1964	CIRM-CFBP^b^
RS105	China	1992	Fu and Xu, 1997; Song^c^, personal communication
B8–12	China	2007	Song^c^
L8	China	Prior to 1995	Zeng et al., 1995; Yin^d^, personal communication
BXOR1	India	1996	Yashitola et al., 2000
CFBP7331 (also MAI10)	Mali	2003	Gonzalez et al., 2007
CFBP7341 (also BAI5)	Burkina	2009	Wonni et al., 2011
CFBP7342 (also BAI11)	Burkina	2009	Wonni et al., 2014

a *International Rice Research Institute, Los Baños, the Philippines*.

b *International Centre for Microbial Resources – French Collection of Plant-associated Bacteria*.

c *Nanjing Agricultural University, Nanjing, China*.

d *Temasek Laboratories, Singapore*.

### Xoc genome sequencing and assembly and TAL effector sequence parsing

Genomes were sequenced using single molecule, real-time (SMRT) technology (Eid et al., 2009) and assembled *de novo* using HGAP (Chin et al., 2013) v3.0 (Pacific Biosciences, Menlo Park, CA) and the PBX toolkit, as described (Booher et al., unpublished). For each strain, 4–7 SMRT cells were used to achieve ~200× coverage. All cells used the XL-C2 chemistry, except for two cells for strain B8–12, which used the P4-C2 chemistry. For RS105 and BXOR1 the large TAL effector gene cluster from the assembly generated using the PBX toolkit was used in place of the cluster as assembled by HGAP, because HGAP partially collapsed (RS105) or expanded (BXOR1) the cluster. This was detected because very long reads mapping to this region indicated the presence of additional (RS105) or fewer (BXOR1) TAL effector genes in the cluster, matching the PBX toolkit assembly. TAL effector sequences were extracted and parsed using the PBX toolkit (Booher et al., unpublished). The genomic coordinates of all TAL effector genes in each strain and the corresponding RVD sequences are given in tab-delimited text in Supplementary Materials, File S1 (Data Sheet 1).

The existence of any small plasmids that might have been excluded during size selection for the PacBio sequencing was tested by DNA isolation and agarose gel electrophoresis, using *X. campestris* pv. vesicatoria strain 85-10 (Thieme et al., 2005) as a positive control, and none were observed (Supplementary Figure S1 in Data Sheet 2). This was carried out as described (Chakrabarty et al., 2010) except that the E.Z.N.A. Plasmid DNA Mini Kit I (Omega Bio-Tek, Norcross, GA) was used for DNA isolation.

### Rice inoculations and RNA sequencing

*Oryza sativa* L. ssp. *japonica* cv. Nipponbare plants to be inoculated were grown in LC-1 soil mixture (Sungro, Bellevue, WA) in PGC15 growth chambers (Percival Scientific, Perry, IA) in trays approximately 60 cm below a combination of fluorescent and incandescent bulbs providing approximately 1000 μmoles/m^2^/s measured at 15 cm, under a cycle of 12 h of light at 28°C and 12 h of dark at 25°C. Plants were inoculated at 2 weeks with bacterial suspensions in 10 mM MgCl_2_ at approximately OD_600_ = 0.4 or with a mock inoculum of 10 mM MgCl_2_, by infiltration using a needleless syringe. For each inoculum, the second and third leaves of each of four plants were infiltrated at 20 contiguous spots per leaf. From each of the eight leaves inoculated with mock inoculum or a single strain, a 12 cm length of the inoculated portion was collected after 48 h and the tissue from all eight leaves pooled together for RNA isolation. For this, RNA was extracted using a modified hot Trizol protocol (Huang et al., 2005) followed by treatment with RNase-free DNase I (Life Technologies, Carlsbad, CA), then purified using the RNeasy MinElute Cleanup kit (Qiagen, Valencia, CA). This experiment was repeated three times for a total of 36 samples. Libraries were prepared with the TruSeq RNA Sample Prep v2 kit (Illumina, San Diego, CA). To perform RNA sequencing (RNA-Seq), samples were indexed and run six per lane on an Illumina Hiseq 2000 following the protocol supplied by the manufacturer for a single-end, 100 cycle run, producing 862.6 million reads across all samples.

### RNA-seq data processing, analysis, and access

Adapters were trimmed from raw reads using the Trimmomatic v0.22 ILLUMINACLIP function (Bolger et al., 2014) for single end reads, with a maximum of two seed mismatches and a palindrome clip threshold of 30 to trim adapter matches with scores of at least 7. Low quality bases were then trimmed from the ends of reads using BRAT trim v2.0.1 (Harris et al., 2010) to remove bases with Phred scores below 20 (Yu et al., 2012). Reads that were shorter than 24 base pairs after trimming were dropped. The 852.2 million remaining reads were aligned to the *Oryza sativa* L. ssp. *japonica* (cv. Nipponbare) reference genome (v.7.0) downloaded from Phytozyme 10 using the MSU Release 7.0 annotation downloaded from the Rice Genome Annotation Project (Kawahara et al., 2013). Alignment was completed using Tophat v2.06 (Kim et al., 2013) except for the reads from one BLS279 replicate. That sample caused Tophat v2.06 to crash and so was instead aligned using Tophat v2.05 (Kim et al., 2013). Transcripts were assembled using Cufflinks v2.1.1 and the same reference annotation used for the alignment (Trapnell et al., 2010; Roberts et al., 2011), and the resulting annotation files were combined using the Cufflinks script Cuffmerge v1.0.0. The number of reads aligned to each gene was determined using HTSeq-count (Anders et al., 2015) from the HTSeq framework version 0.5.4p3 with the command line options “-i ID -t gene -s no,” which unambiguously assigned 739.2 million reads (86% of the raw reads) to a gene. Raw reads and read counts are available through the NCBI Gene Expression Omnibus with accession number GSE67588. Differentially expressed genes were identified using QuasiSeq (Lund et al., 2012) with the quartile normalization method (Bullard et al., 2010). The replicate number was used as a cofactor, and genes were filtered if they did not have an average gene expression of at least 1 within every replicate or did not have at least one read in one control sample or in all non-control samples. After these filtering steps, 26,517 genes remained and were tested for differential expression, using the method of Nettleton et al. (2006) to compute *q*-values from *p*-values. All genes with *q*-values less than or equal to 0.05 were taken as being differentially expressed, regardless of fold change.

### Phylogenetic tree construction and topology comparison

The nucleotide sequences of 31 housekeeping genes were extracted from each genome using AMPHORA (Wu and Eisen, 2008), and then aligned at the codon level in MEGA v6.0 (Tamura et al., 2013) with the MUSCLE alignment algorithm (Edgar, 2004) using default parameters and retaining gaps. The model of nucleotide substitution that best fit these sequences was identified with jModelTest v2.1.7 using the Bayesian information criterion (BIC) and separately the Akaike information criterion (AIC) (Guindon and Gascuel, 2003; Darriba et al., 2012). The best model tested using the AIC was the general time reversible model with invariant sites (GTR + I). The MEGA-estimated proportion of invariant sites was zero, so this reduced to a GTR model. Based on the BIC, the best model tested was the Hasegawa–Kishino–Yano model with invariant sites (HKY + I). Each model was used in MEGA v6.0 with 1000 rounds of bootstrapping to create a maximum-likelihood phylogenetic tree. Since the topologies of the two trees were identical, including bootstrap values, arbitrarily the GTR + I tree was retained.

Orthologous pairs of TAL effectors were identified using the reciprocal best BLAST hits method, ranking BLASTP (Altschul et al., 1997) results by bit score and breaking ties by *E*-value (Moreno-Hagelsieb and Latimer, 2008). BLASTP was run using default parameters. The protein sequences of all TAL effectors as well as the protein sequences of all other genes in the genomes, annotated using RAST (Aziz et al., 2008; Overbeek et al., 2014) and excluding any encoded by reading frames overlapping the TAL effector genes, were included in the search for reciprocal best hits. For every TAL effector, a group containing all orthologs of that TAL effector was created. TAL effectors orthologous to half or fewer of the other TAL effectors in a group were removed from that group, and any TAL effector that was still in two groups after this filtering step was removed from the group in which it had fewer orthologs. This resulted in 39 non-overlapping groups of orthologous TAL effectors. For each strain, corresponding nucleotide sequences were concatenated, and then concatenated sequences were aligned in MEGA v6.0 (Tamura et al., 2013) using the MUSCLE alignment algorithm (Edgar, 2004) using default parameters and retaining gaps. The best model of nucleotide substitution was identified with jModelTest v2.1.7 (Guindon and Gascuel, 2003; Darriba et al., 2012) as above. In this case, the best model, based on either the BIC or the AIC, was the GTR + I model. The MEGA-estimated proportion of invariant sites was zero, so this reduced to a GTR model. A maximum likelihood phylogenetic tree was then created in MEGA using this model with 1000 rounds of bootstrapping. Next, for each ortholog group individually, a phylogenetic tree based on the alignment of the TAL effectors in that group was created in the same way as the tree based on all groups and, for comparison, a phylogenetic tree was created for the strains in each group based on the 31 housekeeping genes in the same way as the housekeeping gene tree for all 10 strains. For each group and for the concatenated alignment of all TAL effector ortholog groups, the one-sided Kishino-Hasegawa test based on pairwise Shimodaira-Hasegawa tests implemented in TREE-PUZZLE (Kishino and Hasegawa, 1989; Shimodaira and Hasegawa, 1999; Goldman et al., 2000; Schmidt et al., 2002) was used to test the null hypothesis that the topology of the maximum likelihood tree created using the TAL effector alignment was not a significantly better fit for those sequence data than the corresponding housekeeping gene tree topology, based on the log likelihood of each topology. Tree topology comparisons were not meaningful for eight ortholog groups containing only two TAL effectors each or for one group containing only six TAL effector sequences that were identical at the nucleotide level.

The tree based on the rice gene expression changes in response to each strain was made using log_10_ fold change values of the 20,136 genes that had enough reads to be tested for differential expression and were differentially expressed in at least one sample. The tree was built using the function heatmap 0.2 from the R package gplots (Warnes et al., 2015), which uses the standard R function hclust from the stats package (R Core Team, 2013) to perform complete linkage clustering to cluster rows and columns of the heatmap based on the Euclidean distance between them. The tree created using only the known BLS256 TAL effector targets was created in the same way.

### Whole genome alignments and recombination breakpoint identification

Whole genome alignments were created in MAUVE using the progressiveMauve algorithm with default parameters (Darling et al., 2010). A codon based alignment of TAL effector genes created using the MUSCLE alignment algorithm (Edgar, 2004) in MEGA v6.0 (Tamura et al., 2013) was searched for recombination breakpoints using GARD (Kosakovsky Pond et al., 2006a,b) on the Datamonkey webserver (Pond and Frost, 2005; Pond et al., 2005; Delport et al., 2010). The GTR model (identified as REV in the GARD menu) was used for this analysis with default settings because this was the best model of nucleotide substitution based on both the BIC and AIC, identified using jModelTest v2.1.7 (Guindon and Gascuel, 2003; Darriba et al., 2012).

### TAL EBE prediction

After TAL effector sequences were extracted from the 10 Xoc genomes, all unique RVD sequences were identified. For every TAL effector containing aberrant repeats of the lengths shown by Richter et al. (2014) to accommodate a single nucleotide deletion at the corresponding position in the DNA, a new TAL effector was artificially added to the list with that repeat missing from the RVD sequence. The original TAL effector was not removed. No TAL effectors with multiple aberrant repeats were observed. TAL effector repeats of atypical lengths other than those characterized by Richter et al. were treated as normal repeats. TAL effectors with fewer than 11 RVDs, which are likely non-functional (Boch et al., 2009), were not included. TAL effector sequences encoded by pseudogenes, characterized by an early stop codon, a frameshift mutation, and or a large insertion that resulted in the absence of any and all matches to the NLS consensus sequence (K-K/R-X-K/R) (Garcia-Bustos et al., 1991; Van Den Ackerveken et al., 1996) or the lack of a stretch of 35 amino acids with at least 80% sequence identity to the acidic activation domain (Zhu et al., 1998; using BLS256 Tal1c as the reference), were also excluded from binding site predictions.

For the retained TAL effectors, EBEs were predicted using the TALE-NT 2.0 Target Finder tool (Doyle et al., 2012). Based on the RVD binding preferences determined by Yang et al. (2014), we updated our local version of Target Finder to treat HH as NH but left treatment of other previously uncharacterized RVDs unchanged, i.e., as having equal affinity for all nucleotides. Predictions were carried out using the rice promoterome, defined as the 5′ UTR (if annotated) plus 1000 base pairs upstream of the transcriptional start site of each transcript; output for a gene includes all unique EBEs predicted in the promoter of any transcript of that gene. Promoters were retrieved from the *Oryza sativa* L. ssp. *japonica* (cv. Nipponbare) reference genome (v.7.0) downloaded from Phytozyme 10 using the MSU Release 7.0 annotation downloaded from the Rice Genome Annotation Project (Kawahara et al., 2013). Predicted EBEs were filtered with a previously developed machine learning classifier. To use the classifier on these large data sets, it was first retrained with transcriptional and translational start site locations from the genome annotation instead of manually curated locations based on EST support as in Cernadas et al. (2014). See Supplementary Table S1 in Data Sheet 2 for updated performance statistics and GitHub, https://github.com/kwilkins226/TALEffectorClassifier, for a Weka model file containing the classifier used here and a script to generate classifier input files from Target Finder output. Only EBEs assigned a probability of being true greater than or equal to 0.5 were included in lists of “passed” binding site predictions.

## Results and discussion

### Whole genome sequences of and rice transcriptional responses to 10 diverse Xoc strains

Based on a Southern blot of *Bam*HI-digested genomic DNAs of 34 geographically diverse strains using a TAL effector gene probe (Supplementary Figure S2 in Data Sheet 2), we selected nine strains in addition to BLS256 (Table 1) that were broadly representative of the restriction fragment length polymorphism observed, and performed SMRT, whole genome sequencing (NCBI BioProject number PRJNA283315). Only one strain, CFBP2286, carries any plasmid (see also Supplementary Figure S1 in Data Sheet 2).

In addition, we determined rice gene expression changes in response to each strain by RNA-Seq of leaf tissue 48 h after inoculation (NCBI Gene Expression Omnibus accession GSE67588).

### TAL effector sequences of the 10 Xoc strains

Across the 10 sequenced strains, 250 TAL effector genes and eight pseudogenes with recognizable TAL effector repeat regions were identified, all chromosomal. One TAL effector gene per strain, including *tal2h* of BLS256, encodes a TAL effector with no activation domain. These each show 95% or greater amino acid sequence identity throughout the C-terminal region (downstream of the CRR) to Tal2h. The gene in the African strain CFBP7342 is a pseudogene disrupted by an insertion sequence element, and the genes in the other two African strains, CFBP7331 and CFBP7341, have only six RVDs each, but in all other strains the genes display intact CRRs of 18 or more repeats. Seven of them contain one atypical-length repeat 28 amino acids in length, not matching one of the types characterized by Richter et al. (2014) as being able to accommodate a single base deletion at the corresponding location in the EBE (File S1, Data Sheet 1). Although BLS256 Tal2h was originally assumed to be nonfunctional due to its truncated C-terminus and lack of activation domain (Bogdanove et al., 2011), the broad conservation of this TAL effector variant suggests that it may serve an important function, perhaps as a virulence factor (Booher et al., unpublished). The use of engineered TAL effectors missing activation domains as transcriptional repressors in eukaryotes suggests a mechanism by which Tal2h could influence host gene expression (Blount et al., 2012; Werner and Gossen, 2014). Notably, the truncated C-terminus retains a predicted nuclear localization signal. Similarly, the atypical repeats of lengths not characterized by Richter et al. could be nonfunctional, but broad conservation suggests otherwise.

The remaining 241 structurally complete TAL effectors collectively represent 98 unique binding specificities, based (solely) on RVD composition and presence or absence of atypical-length repeats (File S1, Data Sheet 1). Six of the 241 have a single atypical-length repeat matching one of the types characterized by Richter et al. (2014) and none has more than one. Four repeats of 36 amino acids each are also observed in four different TAL effectors. The distribution of RVDs observed in the TAL effector sequences (excluding those encoded in pseudogenes) is similar to that of previously sequenced *Xanthomonas* TAL effectors (Boch and Bonas, 2010), with 75% being HD, NN, NG, or NI (Supplementary Figure S3 in Data Sheet 2). Only 40 of the total 1916 RVDs are of types not assigned any specificity in the TALE-NT 2.0 Target Finder binding site prediction tool (Doyle et al., 2012). Among these are two RVDs, QD and HY, each occurring once, that have not been reported previously in any *Xanthomonas* TAL effector.

### TAL effector conservation and known TAL effector targets upregulated by the Xoc strains

**Figure 1:**
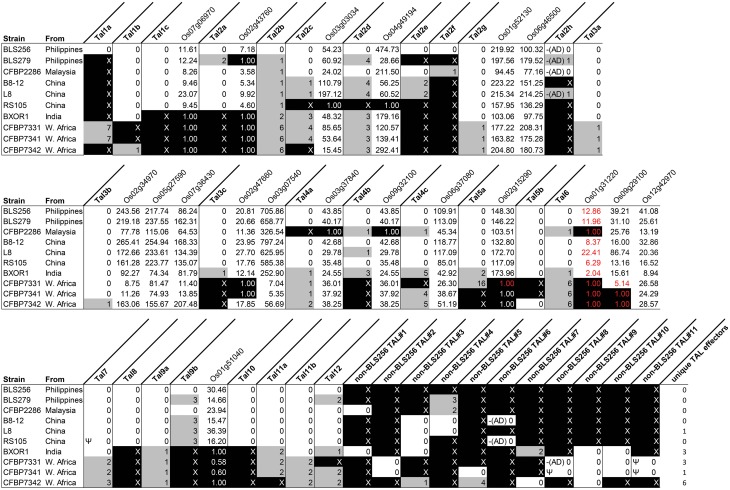
**TAL effector content of diverse Xoc strains in relation to BLS256, and shared upregulation of BLS256 TAL effector targets**. Each column labeled in bold font represents a group of orthologous TAL effectors, identified by finding reciprocal best BLAST hits. Columns are labeled according to the BLS256 ortholog, or if absent, by the designation “non-BLS256 TAL,” numbered arbitrarily. In these columns, the number of BSRs by which each TAL effector in the group (by strain) differs from the reference TAL effector for that group is given. The reference is the BLS256 TAL effector if present and the TAL effector with the most conserved BSR sequence otherwise. Gray fill indicates at least one difference. An “X,” with black fill, indicates absence of an ortholog. “Ψ” indicates that the TAL effector sequence derives from a pseudogene. “–(AD)” indicates a C-terminus like that of BLS256 Tal2h, missing the activation domain. Columns labeled in regular font with a rice gene name (locus ID) show the fold upregulation of that gene by each strain, with black fill indicating genes that are not significantly upregulated. The genes are grouped by the BLS256 TAL effector that upregulates them, immediately to the right of the column for that TAL effector. Fold change values in red font mark instances in which the gene promoter has no predicted EBE that passes a machine learning filter for the BLS256 TAL effector ortholog present in the strain represented in that row.

To assess conservation of TAL effector sequences across strains, we grouped TAL effectors by apparent orthology (descent from the same ancestral gene with no duplication events; see Materials and Methods) (Figure 1). To ascertain potential variation in targeting specificity within groups, we then recorded the number of BSR differences among the TAL effectors in each group, using the BLS256 TAL effector in the group as a reference, if present, or the TAL effector that was conserved exactly in the most strains otherwise (Figure 1). For orthologs of the 13 BLS256 TAL effectors with one or more known targets (Cernadas et al., 2014), we attempted to infer function and targeting specificity by asking whether the ortholog is predicted to bind the promoter for each corresponding target, and whether each target was upregulated by the corresponding strain in our RNA-Seq experiment. Whenever a BLS256 TAL effector is perfectly conserved at the BSR level in another strain, that strain upregulated the known target(s) (Figure 1). For imperfect matches, results were mixed. With one exception discussed below, every BLS256 TAL effector ortholog with no more than six BSR differences has in the promoter of each corresponding BLS256 target gene a predicted EBE that passes a machine learning filter for predicting functionality (see Materials and Methods), and for 99% of these, the target is upregulated. The exception is the BLS256 Tal6 ortholog group. Neither Tal6 nor any of its orthologs has an EBE that passes the filter in the confirmed Tal6 target *Os01g31220*. The reason for this is not clear. Nonetheless, since target activation by BLS256 in all cases was shown to be TAL effector dependent, for any ortholog the presence of a predicted EBE and upregulation of the corresponding target is strong evidence of activation by that ortholog in each case. Where this is the case, the ortholog likely serves the same function as the BLS256 TAL effector, but we cannot exclude the possibility that non-identical orthologs target different genes distinct from the BLS256 TAL effector target(s).

Five TAL effector groups have an ortholog from every strain that is neither pseudogenized nor more than two BSRs diverged from the others. These include the group containing BLS256 Tal2g, a virulence factor that activates the *S* gene *OsSULTR3;6* (Cernadas et al., 2014), and indeed, all 10 strains activate that gene. That all strains use the same TAL effector to upregulate this major *S* gene might reflect a targeting constraint at the promoter that could make modification or deletion of the EBE an effective strategy for durable resistance, one that the pathogen might not readily overcome by targeting a different sequence in the promoter. Another group contains BLS256 Tal11b, a TAL effector of which a knock-out mutant showed reduced virulence on Nipponbare (Cernadas et al., 2014) but for which a target has yet to be identified. The remaining three TAL effector groups include Tal3a, Tal3b, and Tal9a of BLS256, respectively. Although no non-redundant role in virulence for these three TAL effectors was detected based on mutant phenotypes in Nipponbare in a growth chamber (Cernadas et al., 2014), their conservation suggests that they might perform an important function in other rice genotypes or in the field.

While conservation of a TAL effector suggests that the TAL effector is important, conservation of TAL effector target upregulation in the absence of TAL effector conservation suggests that the target gene is important. As an example, Tal3c of BLS256, though conserved in all non-African strains, is absent from the three African strains, yet its two known targets, *Os02g47660* and *Os03g07540* (which are upregulated by all the non-African strains), are upregulated, respectively, by CFBP7342 and by all three African strains (Figure 1). Because the activation of these genes by BLS256 depends on a TAL effector (Cernadas et al., 2014), their upregulation by the African strains seems likely to as well. Consistent with that hypothesis, a TAL effector present only in CFBP7342 has a predicted EBE in the promoter of *Os02g47660* and several predicted EBEs in the promoter of *Os03g07540* that pass the machine learning classifier. The *Os02g47660* EBE does not overlap the Tal3c EBE, but one of the *Os03g07540* EBEs does. Two more TAL effectors, found in all three African strains (one only in the three African strains and the other also in BXOR1), both have multiple, predicted, passing EBEs in *Os03g07540*. One predicted, passing EBE for each of these TAL effectors overlaps the Tal3c EBE by 10 base pairs, and the other overlaps it completely (Supplementary Figure S4 in Data Sheet 2). In addition to pointing to new, strong candidate TAL effector-target pairs, these observations suggest that *Os03g07540*, the Tal3c target upregulated by all strains though apparently by different TAL effectors, is important in bacterial leaf streak. This is further supported by the fact that *Os03g07540* encodes a member of the bHLH protein family, which includes *UPA20*, the *S* gene target of *X. campestris* pv. vesicatoria TAL effector AvrBs3 in pepper (*Capsicum annuum*) (Kay et al., 2007). A *tal3c* mutant strain of BLS256 was not significantly less virulent in a growth chamber using artificial inoculation, but again, it seems likely that such an assay fails to detect differences that would be significant under field conditions.

### Relationships of TAL effectors across strains in comparison to housekeeping genes

**Figure 2:**
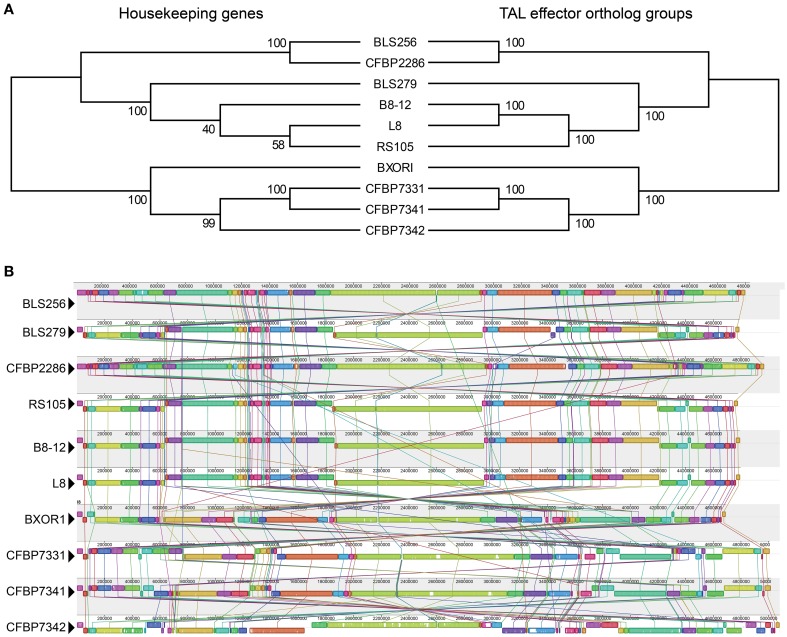
**Phylogenetic relationships based on housekeeping genes vs. TAL effectors, and whole genome alignments of the Xoc strains**. **(A)** Maximum likelihood trees, created using concatenated alignments of the nucleotide sequences of either 31 housekeeping genes or the TAL effectors in each of the 39 ortholog groups shown in Figure 1. Bootstrap values from 1000 replicates are given at the nodes. **(B)** Alignment of the whole genome of every strain, generated using progressiveMauve (Darling et al., 2010). Colored blocks represent regions of uninterrupted homology, connected between genomes by matching colored lines. The height of the colored bars within each block indicates relative conservation on average across all genomes. Homologous regions above the centerline are in the same orientation as they are in BLS256 and those below are inverted.

Conservation of TAL effector binding specificities within the Asian and African groups is high. As a point of reference, the four sequenced Xoo strains (Lee et al., 2005; Ochiai et al., 2005; Salzberg et al., 2008; Booher et al., unpublished), all from Asia, in pairwise comparisons share at most 36% of their TAL effectors and on average only 21% (as a percentage of whichever strain has more TAL effectors, and based on perfect identity of BSR sequences). Between every pair of Asian Xoc strains, a minimum of 25% and on average 57% of TAL effectors are conserved. For the African Xoc strains, the minimum is 32% and the average 51%. Across all the Xoc strains, the average is 33%, but there is clear distinction between the Asian and African groups, with only BXOR1 sharing more than 10% of its TAL effectors with any of the African strains. The comparatively high level of TAL effector conservation within the two Xoc groups could be the result of purifying selection or a relative lack of diversifying selection, but could in part reflect broad dissemination of TAL effector genes via horizontal gene transfer. To address this possibility, we generated and compared two phylogenetic trees (Figure 2A), one based on the sequences of 31 housekeeping genes that are recalcitrant to horizontal gene transfer (Jain et al., 1999; Wu and Eisen, 2008) and the other based on the groups of TAL effector orthologs described above. The two trees are nearly identical, but the tree created using the TAL effector orthologs is a significantly better fit for the TAL effector ortholog alignment than the tree created using the housekeeping genes (Kishino–Hasegawa test *p* = 0.047). This result indicates that TAL effectors may have been horizontally transferred among these strains, though probably infrequently given the similar overall topologies of the two trees. Phylogenies based on individual TAL effector ortholog groups support this conclusion, with only six of thirty testable ortholog groups yielding a TAL effector-based topology that is a significantly better fit for the TAL effector alignment than is the topology of a housekeeping gene-based phylogeny (Supplementary Figure S5 in Data Sheet 2).

For each of those six groups, represented by BL256 Tal2b, Tal2c, Tal2g, Tal4b, and Tal12, and by B8-12 Tal5e (non-BLS256 Tal#4 ortholog group in Figure 1 and Supplementary Figure S5 in Data Sheet 2), the genomic context of each TAL effector gene represented in the group, specifically the local spatial relationship to other TAL effector genes, is conserved across the genomes of the strains in the group (Supplementary Figure S5 in Data Sheet 2 and **Figure 4**). This suggests that either clusters of TAL effector genes were transferred or individual TAL effector genes were transferred to the same locations within those clusters. This second possibility seems unlikely given the overall high sequence similarity across TAL effector genes and often their flanking DNA, which should result in integration of a horizontally transferred TAL effector gene into any cluster randomly. Therefore, if the TAL effector genes in the six groups were horizontally transferred, neighboring TAL effector genes likely were as well. We saw no evidence for this however. For the BLS256 Tal4b and Tal12 groups, TAL effectors encoded by genes on either side yielded tree topologies that fit the TAL effector alignments no better than the housekeeping tree-based topologies did. Similarly, for the groups represented by BLS256 Tal2b, Tal2c, and Tal2g, which are encoded in the same cluster, and for the group represented by B8-12 Tal5e, which is encoded in the orthologous cluster in each of the four genomes in which it occurs, in the same location, TAL effectors encoded by intervening genes in the cluster (Tal2d, Tal2e, and Tal2f in BLS256) likewise showed no evidence of horizontal transfer (the Tal2f group represents only two strains and was therefore not meaningful). Thus, for these six groups, either the difference between the TAL effector-based topologies and the housekeeping gene-based topologies is not due to horizontal gene transfer, or the lack of difference for the groups representing flanking or intervening genes is in each case a false negative.

**Figure 3:**
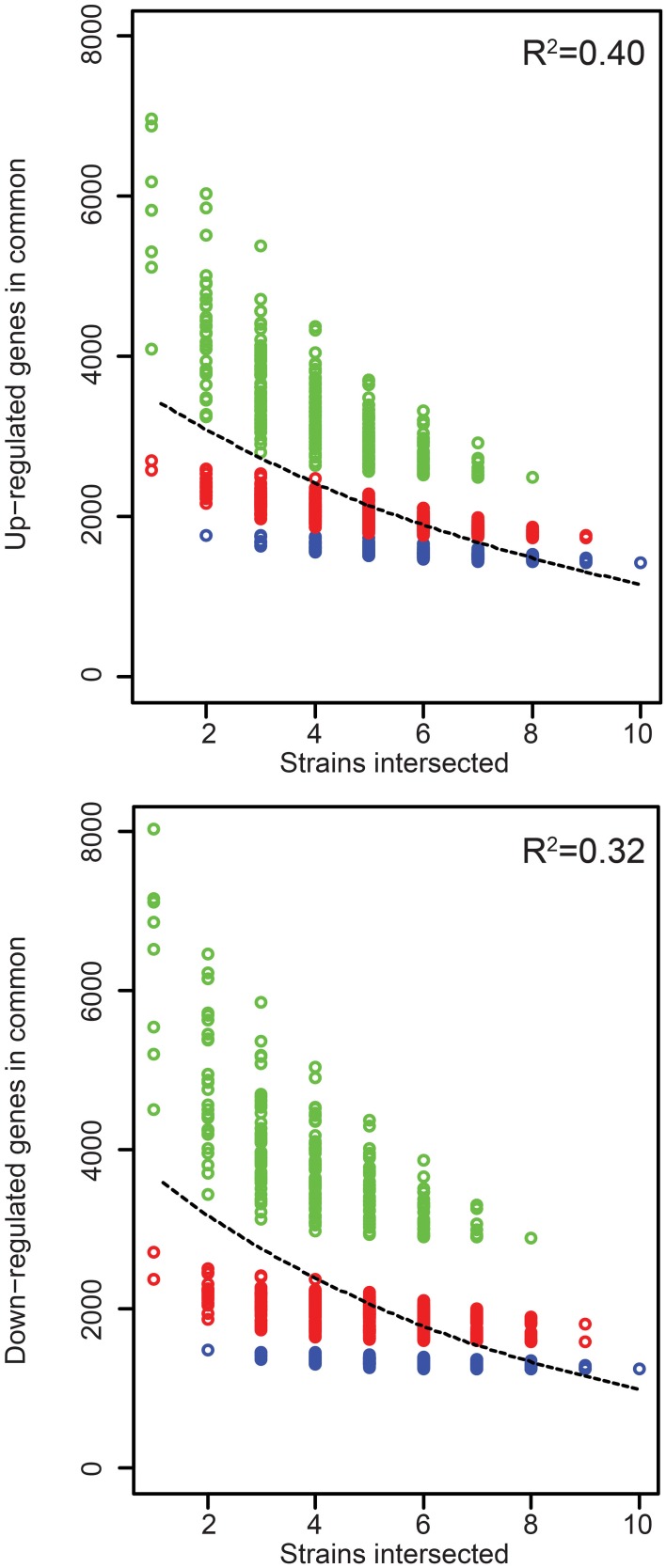
**Rice gene expression changes induced in common by the Xoc strains**. The number of genes up- or down- regulated in rice (cv. Nipponbare, at 48 h after syringe infiltration) in common by each of *n* strains across all possible groups of strains is plotted as a function of *n*. The least-square fit of an exponential decay function is shown as a dashed line, with *R*^2^ values for the fit displayed in the upper-right of each graph. CFBP2286 and BXOR1 induced the fewest gene expression changes (up or down), and this is reflected in stratification of the points in each plot. To make this apparent, groups containing only one of these strains are shown in red and groups containing both in blue.

Consistent with the high levels of Xoc TAL effector conservation, the Xoc genomes overall are highly structurally conserved. Only 53 recombination breakpoints are required to explain the genome rearrangements observed among the strains (Figure 2B), and no breakpoints detectable by GARD (Kosakovsky Pond et al., 2006b) occur within a TAL effector gene. With minor exceptions that likely arose from exchange between two TAL effector genes by double cross-over, or in some cases local rearrangements associated with insertion sequence elements, all apparent orthologs reside in conserved contexts within their respective genomes (Figure 3).

### TAL effector content in relation to rice gene expression changes

**Figure 4:**
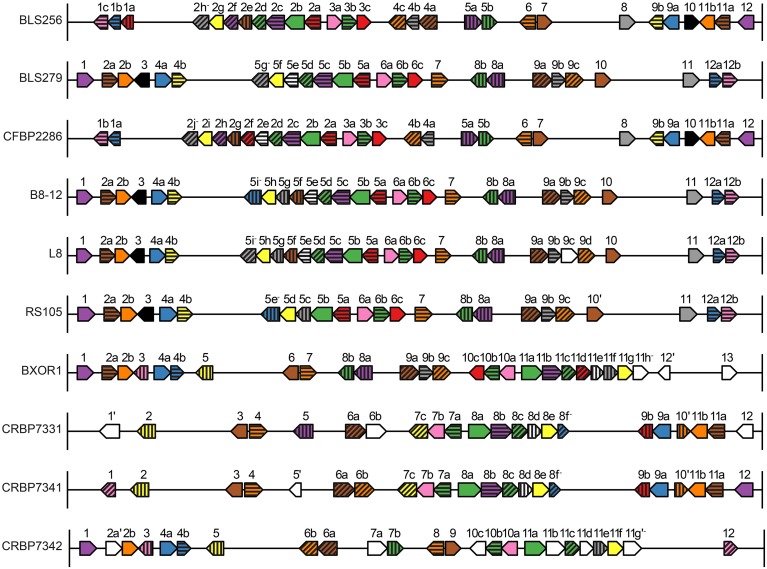
**Identity, order, and orientation of TAL effector genes across the Xoc strains**. Each arrow represents a TAL effector (*tal*) gene and points in the 5′ to 3′ direction. The genes are to scale relative to one another, as are the intergenic regions, but the genes have been magnified relative to the rest of the genome. Arrows of the same color and pattern represent orthologs. The same color or pattern alone does not indicate any particular relationship. Genes are numbered and lettered according to Salzberg et al. (2008). An apostrophe indicates a pseudogene, and a minus sign indicates that the encoded TAL effector has a BLS256 Tal2h-like C-terminus, lacking the activation domain.

Because even similar TAL effectors may target different sets of host genes and because different TAL effectors may share targets (Yang et al., 2006; Antony et al., 2010; Römer et al., 2010; Yu et al., 2011; Streubel et al., 2013; Richter et al., 2014), differences in TAL effector content between strains by itself may not be predictive of differences in the changes to rice gene expression each strain causes. To examine this, we compared the host gene expression changes in response to all the strains in relation to their TAL effector content. The RNA-Seq results we obtained show that the Xoc strains on average upregulate 5152 rice genes, but only 1437 (28%) of these are upregulated by every strain (Table 2). The number of genes downregulated by each strain is similar, 5608 genes on average, with 1248 (22%) of these downregulated in common. Every gene expression change uniformly required for the development of bacterial leaf streak of rice by definition is represented among the genes up- or down- regulated by all 10 strains. The relatively small number of these genes up- or down- regulated in common might be explained in part by the exclusion of any gene expression change that serves a required function but is substituted for in the case of some strains by a distinct induced change. Upregulation of different *SWEET* gene family members by different TAL effectors of different Xoo strains in bacterial blight of rice is an example of such essential but interchangeable TAL effector induced changes to host gene expression (Yang et al., 2006; Antony et al., 2010; Römer et al., 2010; Yu et al., 2011; Streubel et al., 2013; Richter et al., 2014). Incidentally, the list of gene expression changes induced in common by every strain almost certainly contains a number of non-essential changes: plotting the number of shared up- and down-regulated genes among all possible combinations of the strains reveals that an exponential decay function fits the data poorly (Figure 4); in other words, the rate at which the number of shared gene expression changes decreases does not decrease as more strains are added, up to the 10 total.

**Table 2:** **Numbers of TAL effectors, induced rice gene expression changes, and target predictions for each Xoc strain**.

**Strain or value**	**TAL effectors^a^**	**Upregulated genes^b^**	**Downregulated genes^c^**	**Predicted EBEs^d^**	**Passed EBEs^e^**	**Predicted candidate genes^f^**	**Passed candidate genes^g^**
B8-12	27(1)	6966	7112	55,980	23,602	6966	3930
BLS256	27(1)	6881	8043	55,980	24,980	6881	3976
BLS279	25(1)	6191	6872	55,980	23,539	6191	3538
BXORI	25(2)	2592	2373	55,981	30,117	2592	1771
CFBP2286	27(1)	2708	2713	55,980	25,581	2708	1609
CFBP7331	19(3)	5117	5554	55,980	32,080	5117	3623
CFBP7341	19(3)	5306	5213	55,980	31,977	5306	3715
CFBP7342	22(2)	5830	6531	55,983	45,973	5830	5065
L8	26(3)	5835	7157	55,980	24,994	5835	3479
RS105	22(2)	4093	4509	55,980	21,604	4093	2186
Average	24	5152	5608	55,980	28,445	5152	3289
Shared^h^	5	1437	1248	55,979	16,425	1437	648

a *The number in parentheses is the number of pseudogenes*.

b *The number of genes upregulated in rice (cv. Nipponbare) leaves at 48 h after syringe infiltration of the strain relative to mock inoculated leaves (q < 0.05)*.

c *The number of genes downregulated in rice (cv. Nipponbare) leaves at 48 h after syringe infiltration of the strain relative to mock inoculated leaves (q < 0.05)*.

d *The number of genes with at least one predicted EBE in the promoter*.

e *The number of genes with at least one EBE in the promoter that passes the machine learning classifier filter*.

f *The number of upregulated genes with at least one predicted EBE in the promoter*.

g *The number of upregulated genes with at least one EBE in the promoter that passes the machine learning classifier filter*.

h *Shared TAL effectors are defined as having no more than two BSR differences*.

**Figure 5:**
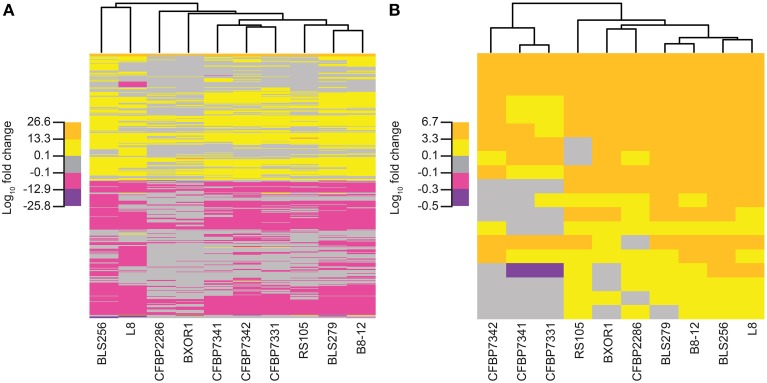
**Relationships of the Xoc strains based on the rice gene expression changes each induces**. Trees were created using **(A)** for each strain, fold change values for all genes that are significantly differently expressed (*q* ≤ 0.05) at 48 h following syringe infiltration, relative to mock (see Methods) and **(B)** only the fold change values for the known targets of BLS256 TAL effectors. Each tree was generated by complete linkage clustering based on the Euclidean distance between vectors of log_10_ fold change. For each tree, five subdivisions of the log_10_fold change values observed in the data for that tree were used: the minimum, half of the minimum, the maximum, and half of the maximum.

Overall, relationships among the rice transcriptional responses to the 10 Xoc strains, shown as a tree in Figure 5A (see Materials and Methods), do not match those observed for the TAL effectors (Figure 2A). Of particular note are the Asian strains, which cluster distinctly in the TAL effector based tree, but not in the tree based on rice gene expression changes. This greater conservation of induced host gene expression changes than TAL effector content may be due to TAL effector-independent changes, and or convergent evolution having resulted in diverse TAL effectors sharing targets, as suggested by the targets of BLS256 Tal3c and exemplified by the *SWEET* genes in bacterial blight, discussed above. Perhaps not surprisingly, however, when only the expression changes of the known targets of BLS256 TAL effectors are examined (Figure 5B), the clustering more closely recapitulates the tree based on the orthologous TAL effector groups (Figure 2A).

### Toward identification of new TAL effector targets in bacterial leaf streak of rice

TAL effectors often determine whether an interaction leads to disease or to successful plant defense. In addition to augmenting the promoter of an *R* gene with EBEs for conserved TAL effectors to broaden its effectiveness (Hummel et al., 2012; Zeng et al., 2015), genetic manipulation of host *S* gene promoters to prevent activation, through breeding or by genome editing, can be a successful disease control strategy (Yang et al., 2006; Li et al., 2012b). For broad effectiveness and durability, identification of genes widely targeted by strains within and across pathogen populations, either by conserved TAL effectors or distinct ones in different strains, is an important starting point. Toward this goal, we first sought to identify candidate TAL effector targets for each of the 10 Xoc strains by predicting whether any of the genes upregulated by a strain contains a possible EBE in its promoter for any of the TAL effectors of that strain. For this, we used the Target Finder tool of TALE-NT 2.0 with a score ratio threshold of 3.0 (Doyle et al., 2012). For any TAL effector with an atypical-length repeat of a type characterized by Richter et al., we ran predictions using the complete RVD sequence as well as that sequence with the RVD of the repeat left out. We treated repeats of other atypical lengths as typical repeats. We did not do predictions for TAL effectors with the truncated Tal2h-like C-terminus, reasoning that if they are functional (1) they might act by targeting outside the promoter (Politz et al., 2013), and (2) their targets might include genes not differentially expressed in comparison to the mock control used in the RNA-Seq experiment, but instead prevented from being otherwise activated by the pathogen. Somewhat surprisingly, every gene upregulated by any strain has a predicted EBE for at least one TAL effector of that strain, meaning that the predictions provide no additional information beyond the RNA-Seq data. In fact, Target Finder predicts an EBE for at least one TAL effector per strain in nearly all of the 55,986 gene promoters in the MSU7 (Kawahara et al., 2013) annotation (Table 2). Consequently, in order to identify a testable list of candidate TAL effector targets from this and like studies of wild type strains with multiple TAL effectors, either more specific (fewer false positive) EBE predictions, or methods to rank and filter candidate genes are essential.

A modified version of the machine learning classifier from Cernadas et al. (2014) (see Methods) appears to perform well as a way to filter candidate genes. If instead of starting with upregulated genes, the whole genome is examined, using only EBEs passing this classifier reduces the number of candidate genes by 36% across all samples, compared to not using the classifier (Table 2). Of the resulting set of genes with passing EBEs, 12% are upregulated, a significant increase from the 9% of all genes with predicted EBEs that are upregulated (*p*<0.01). This enrichment suggests that the filtering is beneficial and could be applied to the list of candidates from the upregulated gene set. Having RNA-Seq data for the host interacting individually with each of multiple strains for which the TAL effector content is known enables a second, independent method of filtering (the machine learning classifier does not use the RNA-Seq data as input), eliminating any candidate target that is not upregulated by every strain that contains the corresponding TAL effector. All known BLS256 TAL effector targets pass this filter. Though expected, this result provides some assurance that requiring a candidate gene to be upregulated by all strains containing the relevant TAL effector will not eliminate true TAL effector targets from consideration. The two filters discussed here, the classifier and the shared TAL effector-shared target criterion, can be used together or independently. Supplementary Table S2 (Data Sheet 3) provides a list of candidates for the five TAL effectors shared, with no more than two BSR differences, by all 10 strains, generated using both filters together. These lists and others that could be generated for geographical subsets of these strains represent promising starting points for future experimental analyses to identify additional TAL effector targets, including new BLS *S* genes.

## Conclusion

As discussed in the introduction, new strategies centered on TAL effectors and their targets show great promise for control of plant diseases in which TAL effectors play determinative roles (Boch et al., 2014). One of these, amending an *R* gene promoter with one or more additional EBEs, for breadth and durability requires using EBEs that correspond to conserved TAL effectors or to a minimal set of representative TAL effectors so that it will trap the diversity of strains in a population. Another, integrating or engineering an allele of a major *S* gene that is immune to activation due to disruption of its EBE(s), requires identifying an *S* gene on which the strains in a population uniformly depend. Characterizing population level TAL effector conservation addresses the first of these requirements, and assessing host genome-wide expression data alongside TAL effector sequences helps address the second by enabling identification of conserved and potentially important candidate TAL effector targets. The analyses we present in this paper of TAL effector sequences from 10 diverse Xoc strains (from eight genome sequences we present here and two we determined previously), and RNA-Seq data we captured for rice responding to each strain, are a significant advance toward these objectives for the increasingly globally important rice disease bacterial leaf streak.

Our RNA-Seq results are consistent with the microarray-based study of BLS256-inoculated rice we published previously (Cernadas et al., 2014): all TAL effector targets identified in that study are upregulated in the RNA-Seq data for all strains that contain a TAL effector with the corresponding RVD sequence. However, because RNA-Seq is more sensitive than hybridization to a microarray, and because, for reasons outlined below, we predicted a greater number of binding sites per TAL effector, on average, than we previously predicted for the BLS256 TAL effectors (Cernadas et al., 2014), here we were able to identify, with filtering, candidate targets for all of the 27 intact BLS256 TAL effectors except Tal2b (which is not, as it was originally described, a pseudogene; Booher et al., unpublished), in contrast to the 19 TAL effectors for which we were able to identify candidate targets using the microarray data, without filtering (Cernadas et al., 2014).

The greater number of EBE predictions here is due, in part, to the presence of more uncharacterized RVDs in this larger collection of TAL effectors. Among the BLS256 TAL effectors, Tal2g contains the only two uncharacterized RVDs. In our previous work, we replaced these with RVDs of known specificity that shared the same BSR (Cernadas et al., 2014). The TAL effectors in the present study include 40 previously uncharacterized RVDs, and based on recent experimental results (Yang et al., 2014; Miller et al., 2015), we chose to replace only some of these with observed RVDs of known specificity. The impact of uncharacterized RVDs on binding site prediction specificity is evident in the filtered candidate gene lists for the five highly conserved groups of orthologous TAL effectors. The filtered candidate gene lists for four of the ortholog groups contain fewer than 11 genes. The TAL effectors in these ortholog groups contain no uncharacterized RVDs. The gene list for the fifth group, which includes Tal2g, has 521 candidate genes that pass both filtering steps. Each TAL effector in that group contains two uncharacterized RVDs. The large number of candidate genes identified for this group almost certainly results from the binding site prediction algorithm treating uncharacterized RVDs as having equal affinity for all four nucleotides.

Another reason for the greater number of candidates in this study is our having considered all potential EBEs in a promoter, instead of just the best scoring one. This allows for the possibility that a poorer scoring EBE that is closer to the transcriptional start site is more likely to activate a gene than a better scoring EBE that is at a distance, and results in more candidates passing the machine learning filter. We also redefined the promoter as the 5′ UTR plus 1000 bp upstream of the transcriptional start site, instead of just the latter, which was used previously. This allows for the possibility that some TAL effectors activate their targets by binding within the 5′ UTR of the basal transcript and resetting the transcriptional start site. Finally, in our previous study (Cernadas et al., 2014), we determined the EBE score cutoff for each TAL effector individually using the distribution of the best predicted binding sites in each gene in the rice genome. In the present study, we fixed the score cutoff at three times the best possible score, reflecting the current Target Finder algorithm.

With the greater sensitivity of RNA-seq and the prediction parameters we used, absent a method to more specifically predict EBEs, the filtering is essential to obtain a testable number of candidate targets from RNA-Seq studies using wildtype strains with multiple TAL effectors. Generating individual TAL effector knockout strains for comparison to the wild type is desirable, but not always feasible. Comparison across distinct wildtype strains is a powerful alternative. In the case of BLS256 Tal3c target *Os03g07540*, this approach enabled us to distinguish this gene from among the several targets of that TAL effector as potentially important, because it was the only Tal3c target upregulated by all strains, ostensibly due to being targeted by a TAL effector distinct from Tal3c in some strains.

In light of our overall results, TAL effector-centered strategies for control of BLS appear promising. We observed that within geographic regions, Xoc TAL effectors are highly conserved relative to Xoo TAL effectors. They are not disrupted by breakpoints that lead to large, genomic rearrangements, and they appear rarely or never to have been horizontally transferred within Xoc, consistent with the observation of Ferreira et al. (2015) that Xoc TAL effector genes do not localize with TnX*ax1*short inverted repeat sequences to form mobile cassettes, unlike TAL effector genes in other *Xanthomonas* species. Moreover, we identified five Xoc TAL effectors that are shared by all 10 sequenced strains with no more than two BSR differences. As discussed above, one of these groups, the BLS256 Tal2g group, targets the major *S* gene *OsSULTR3;6*, indicating that edited or naturally occurring alleles lacking the corresponding EBE might be broadly effective for disease control. An *R* gene amended with EBEs to capture all five groups also seems likely to be broadly effective and durable.

Finally, the conserved TAL effector groups represent footholds for further dissecting the functions of TAL effectors and their targets. The fact that one of the groups contains BLS256 Tal11b, for which a mutant strain showed reduced virulence but for which no target was identified (Cernadas et al., 2014), supports the conclusion that Tal11b and the orthologs are important but may act in a non-canonical way, such as downregulating a gene by binding to its 5′ UTR. The three groups for which no virulence contribution of the BLS256 TAL effector was observed in the growth chamber assay of Nipponbare plants, as discussed, may exemplify TAL effectors with host genotype-specific virulence contributions, or functions not measured by our virulence assay but important under field conditions. These might include contributing to the ability of the pathogen to initiate infection from a low titer or to disseminate. Broad conservation of the TAL effectors in each of these cases recommends them for further experimentation to test these possibilities.

## Author contributions

LW, KW, and AB conceived and designed the study. LW and KW carried out the experiments. KW, NB, and AB analyzed and interpreted the data. KW and AB prepared the paper, with assistance from LW and NB.

### Conflict of interest statement

The authors declare that the research was conducted in the absence of any commercial or financial relationships that could be construed as a potential conflict of interest.
