# Supplementary material for: TAL effectors and activation of predicted host targets distinguish Asian from African strains of the rice pathogen Xanthomonas oryzae pv. oryzicola while strict conservation suggests universal importance of five TAL effectors
Source: Front Plant Sci. 2015 Jul 21;6:536. doi: 10.3389/fpls.2015.00536 (PMC4508525; doi:10.3389/fpls.2015.00536)
Supplement: Supplementary file 1 [file DataSheet1.ZIP › FileS1/TALEffectorRVDSequences.pdf]

B8-12

**tal1**	NS HD NI N* NI HG NI NI N* NG HD NN NI NG HD NN NS NN NG NN
**tal2a**	HD NN HD N* NS NN HG NI NI NS NI NG HD NN HD HD NG HG NG
**tal2b**	HD HD HD HD HD NG HD NN HD ND NG NG NN HG ND ND HD NG
**tal3**	HD HG HD HG N* NN NG HD NN HD NG NG NN HD N* NG NG
**tal4a**	NN NS NG NI HD HD NG NN HD NI NG N* N* NG HD HD NS NI NN NG NG
**tal4b**	NI NN NI HD NN NG HD NN HD HG HD HG HG HD HD NG
**tal5i-**	NI ND HG HG HG HG NG HG HG HD HD HD HD NN NN HD HG HH H* H* NN HD H*
**tal5h**	NN NN ND N* NS NS YG NI SN ND HD NG HD NS NN HD NG
**tal5g**	NN HD NI N* NI NI NK HD HD HD HG NI NN HD HD
**tal5f**	NI NN NI NI NI NI N* NI NG NN NN NN NG NS NG HD
**tal5e**	NI HG N* NI SN HD HD HD NN NI NK HD NN NN HD NG
**tal5d**	NI HG N* NN N* HD HG HD NN HD NN HG NG NS N* HD
**tal5c**	HD HD NN NN NG N* HD NI NG HD NG NS HD HA HD N* HD NN HD NN NN HD HD NG NN NG HD
**tal5b**	NI HG NI NG HN NG NN ND HD NG HD NI HG HG HD NN HD HH N* HD NN NN NG HD NN NN NG NN NI NG
**tal5a**	HD HD NC HD NC NG HD HH HD NI NG N* NS N* HD HD NS NI HG HD NG
**tal6a**	NN HD NI NI NG NN NN HD NN NI NI NN NN NN NG NI HD NG
**tal6b**	NI N* NI NI NN N* NI NK HD HD HD NG NI NN HD NN HD NG
**tal6c**	NN HD NI HD NN NG NG HD NN HD N* NN HD HD HD NG
**tal7**	NN HD NI NI NN HA NN NS NS NI HD HA HA HA HD HD HD HA HD N*
**tal8b**	HH NN HD HD NG N* HD NN HD ND N* NG NS NS NN HD HD NG
**tal8a**	NN HD NS NG HD NN HD NI HD NN HD NN HD NN HD NN NN NN NN NN NN NN HD NG
**tal9a**	NI NG NI NG NN NG NN NG HD NN NN HG HD NN NS NN HD HD NG NA NN HD HD HD HD N*
**tal9b**	NS HG NI NG NS NN NG NN NG HD NN HD HD NG
**tal9c**	NS HG NI NG NS NN ND N* NG N* HN NN HD NS NI NN HD HD NG NG HG HD NG
**tal10**	NN HD HD HH NN NG NS NG HD NI NG HD HH NG NG NG HA NG
**tal11**	NN NG HD NG HD HD NI NH HG NI NN NN HD NS NG NN HD NG NG N*
**tal12a**	NI NN NI NI NG HD NG NN NI NN NI NN HD
**tal12b**	HD HD HD HD HD NG HD NN HD NG HG NN HD N* NG NG

BLS256

**tal1c**	HD HD HD HD HD NG HD NN HD NG HG NN HD N* NG NG
**tal1b**	NI NN NI NI NG HD NG NN NI NN NI NN HD
**tal1a**	NN HD NI NK HD HD HD NG NI NN HD HD NG
**tal2h-**	NS ND HG HG HG NG HG HG HD HD HD NN NN HD HG HH H* H* NN HD H*
**tal2g**	NN NN ND N* NS NS YG NI SN HD HD NG HD NS NN HD NG
**tal2f**	HD HD NG NI HG HD HG NI HD NN HD HG HG HG NG NI NG
**tal2e**	NI NN NI NI NI NI N* NI NG NN NN NN NI NS NG NG
**tal2d**	NI HG N* NN N* HD HG HD NN HD NK HG NN NN NG HD
**tal2c**	HD HD NN NN NG N* HD NI NG HD NG NS HD HA ND N* ND NN HD NN NN HD HD N* NN NG HD
**tal2b**	NI HG NI N* HN NG NN ND HD NG HD NI HG HG HD NN HD HH N* HD NN NN NG HD NN NN NG NN NI NG
**tal2a**	HD HD NC HD NC NG HD HH HD NI NG N* NS N* HD HD NS NI HG HD NG
**tal3a**	NN HD NI NI NG NN NN HD NN NI NI NN NN NN NG NI HD NG
**tal3b**	NI N* NI NI NN N* NI NK HD HD HD NG NI NN HD NN HD NG
**tal3c**	NN HD NI HD NN NG NG HD NN HD N* NN HD HD HD NG
**tal4c**	NS HG NI NG NS NN ND N* NG N* HN NN HD NS NI NN HD HD NG NG HG HD NG
**tal4b**	NS HG NI NG NS NN NG NN NG HD NN HD HD NG
**tal4a**	NI NG NI NG NN NG NN NG HD NN NN HG HD NN NS NN HD HD NG NA NN HD HD HD HD N*
**tal5a**	NN HD NS NG HD NN HD NI HD NN HD NN HD NN HD NN NN NN NN NN NN NN HD NG
**tal5b**	HH NN HD HD NG N* HD NN HD ND N* NG NS NS NN HD HD NG
**tal6**	NN HD NI NI NN HA NN NS NS NI HD HA HA HA HD HD HD HA HD N*
**tal7**	NN HD HD HH NN NG NS NG HD NI NG HD HH NG NG NG HA NG
**tal8**	NN NG HD NG HD HD NI NH HG NI NN NN HD NS NG NN HD NG NG N*
**tal9b**	NI NN HN NN NI NG HD NN HD HG HD HG HG HD HD NG
**tal9a**	NN NS NG NI HD HD NG NN HD NI NG N* N* NG HD HD NS NI NN NG NG
**tal10**	HD HG HD HG N* NN NG HD NN HD NG NG NN HD N* NG NG
**tal11b**	HD HD HD HD HD NG HD NN HD ND NG NG NN HG ND ND HD NG
**tal11a**	HD NN HD N* NS NN HG NI NI NS NI NG HD NN HD HD NG HG NG
**tal12**	NS HD NI N* NI HG NI NI N* NG HD NN NI NG HD NN NS NN NG NN

BLS279

**tal1**	NS HD NI N* NI HG NI NI NG NG HD NN NI NG HD NN NS NN NI NN
**tal2a**	HD NN HD N* NS NN HG NI NI NS NI NG HD NN HD HD NG HG NG
**tal2b**	HD HD HD HD HD NG HD NN HD ND NG NG NN HG ND ND HD NG
**tal3**	HD HG HD HG N* NN NG HD NN HD NG NG NN HD N* NG NG
**tal4a**	NN NS NG NI HD HD NG NN HD NI NG N* N* NG HD HD NS NI NN NG NG
**tal4b**	NI NN NI HD NN NG HD NN HD HG HD HG HG HD HD NG
**tal5g-**	NI ND HG HG HG NG HG HG HD HD HD NN NN HD HG HH H* H* NN HD H*
**tal5f**	NN NN ND N* NS NS YG NI SN HD HD NG HD NS NN HD NG
**tal5e**	NI NG N* NI SN HD HD HD NN HD NK HD NN HD HD N*
**tal5d**	NI HG N* NN N* HD HG HD NN HD NN HG NG NS N* HD
**tal5c**	HD HD NN NN NG N* HD NI NG HD NG NS ND HA ND N* ND NN HD NN NN HD HD N* NN NG HD
**tal5b**	NI HG NI NG HN NG NN ND HD NG HD NI HG HG HD NN HD HH N* HD NN NN NG HD NN NN NG NN NI NG
**tal5a**	HD HD HD HD HD NG HD HH HD NI NG N* NS N* HD HD NS NI HG HD NG
**tal6a**	NN HD NI NI NG NN NN HD NN NI NI NN NN NN NG NI HD NG
**tal6b**	NI N* NI NI NN N* NI NK HD HD HD NG NI NN HD NN HD NG
**tal6c**	NN HD NI HD NN NG NG HD NN HD N* NN HD HD HD NG
**tal7**	NN HD NI NI NN HA NN NS NS NI HD HA HA HA HD HD HD HA HD N*
**tal8b**	HH NN HD HD NG N* HD NN HD ND N* NG NS NS NN HD HD NG
**tal8a**	NN HD NS NG HD NN HD NI HD NN HD NN HD NN HD NN NN NN NN NN NN NN HD NG
**tal9a**	NI NG NI NG NN NG NN NG HD NN NN HG HD NN NS NN HD HD NG NA NN HD HD HD HD N*
**tal9b**	NS HG NI NG NS NN NG NN NG HD NN HD HD NG
**tal9c**	NS HG NI NG NS NN ND N* NG N* HN NN HD NS NI NN HD HD NG NG HG HD NG
**tal10**	NN HD HD HH NN NG NS NG HD NI NG HD HH NG NG NG HA NG
**tal11**	NN NG HD NG HD HD NI NH HG NI NN NN HD NS NG NN HD NG NG N*
**tal12a**	NI NN NI NI NG HD NG NN NI NN NI NN HD
**tal12b**	HD HD HD HD HD NG HD NN HD NG HG NN HD N* NG NG

BXOR1

**tal1**	NS HD NI N* NI HG NI NI N* NG HD NN NI NG HD NN NS NN NG NG
**tal2a**	HD NN HD N* NS NN NG NI NI NG HY NG HD NN HD HD NG HG NG
**tal2b**	HD HD HD HD HD NG HD NN HD ND NG NG NN HG ND ND HD NG
**tal3**	HD HG N* NN NG HD NN HD NN NG NN HD N* NG NG
**tal4a**	NN NS NG NI HD HD NG NN HD NI NG N* N* NG HD HD NS NI NN NG N*
**tal4b**	NI NN NI NI NG HD NG NN NI NN NI NN HD
**tal5**	NN HG HD NG HD HD NI NH HG NI NN NN HD NS NG NN HD NG NG NG NG
**tal6**	NN HD HD HH NN NG NS NG HD NI NG HD HH NG NG NG HA NG
**tal7**	NN HD NI NI NN HA NN NS NS NI HD HA HA HA HD HD HD NI HD N*
**tal8b**	HH NN HD HD NG N* HD NN HD ND N* NG NS NS NN HD HD NG
**tal8a**	NN HD NS NG HD NN N* NI HD NS HD NN HD NN HD NN NN NN NN NN NN NN HD NG
**tal9a**	NI NG NI NG NN NG HH NG HD NN NN HG HD NN NS NN HD HD NG NA NN HD HD HD HD N*
**tal9b**	NN HG NI NG NI NN NG NN NG HD NN HD HD N*
**tal9c**	NN HG NI NN NG NN ND N* NG N* HN NN HD NN NI NN HD HD NG NG NG HD N*
**tal10c**	NN HD NI HD NS NG NG HD NN HD N* NN HD HD HD NG
**tal10b**	NI N* NI NI NN N* NI NK HD HD HD NG NI NN HD NN HD NG
**tal10a**	NN HD NI NI NG NN NN HD NN NI NI NN NN NN NG NI HD NG
**tal11a**	NI HG NI HG HN NG NN HD ND NG HD NI HG HG HD NN HD HH N* HD NN NN NG HD NN NN NG NN NI NV
**tal11b**	HD HD NN NN NG N* HD NI NG HD NG NS ND N* HD N* HD NG HD NN NN HD HD NG NN NG HD
**tal11c**	NI NG N* NN N* HD HG HD NN HD NN HG HG NS NG HD
**tal11d**	HD HG N* NN NG NN NI NN NI NN NN HD HG NG HD NG
**tal11e**	HD HG HD NI NN ND HD HD NN NN NI HD
**tal11f**	NN HD NI N* NI NI NK HD HD HD NG NI NN HD HD
**tal11g**	NN NN HD N* NS NS YG NI SN HD HD NG HD NS NN HD NG
**tal11h-**	NI HG HG HG HG NG HD HD HD HD HD NG N* NI NN HD HD H*
**tal12'**	NS HD N* NG HY NG NS NG HD N*
**tal13**	NN NI HN NG NN NN NG NI N* NI NG NN NN HG HD HD NG

CFBP2286

**tal1b**	HD HD HD HD HD NG HD NN HD NG HG NN HD N* NG NG
**tal1a**	NI NN NI NI NG HD NG NN NI NN NI NN HD
**tal2j-**	NS ND HG HG HG NG HG HG HD HD HD NN NN HD HG HH H* H* NN HD H*
**tal2i**	NN NN ND N* NS NS YG NI SN HD HD NG HD NS NN HD NG
**tal2h**	HD HD NG NI HG HD HG NI NI NN HD HG HG HG NG NI NG
**tal2g**	NI NN NI NI NI NI N* NI NG NN NN NN NI NS NG NG
**tal2f**	HD HG N* NN HG NN NI NN NI NN NN HD HG NG HD NG
**tal2e**	NI HG N* NI SN HD HD HD NN HD NK HD HD NN HD NG
**tal2d**	NI HG N* NN N* HD HG HD NN HD NK HG NN NN NG HD
**tal2c**	HD HD NN NN NG N* HD NI NG HD NG NS HD HA ND N* ND NN HD NN NN HD HD N* NN NG HD
**tal2b**	NI HG NI NG HN NG NN ND HD NG HD NI HG HG HD NN HD HH N* HD NN NN NG HD NN NN NG NN NI NG
**tal2a**	HD HD NC HD NC NG HD HH HD NI NG N* NS N* HD HD NS NI HG HD NG
**tal3a**	NN HD NI NI NG NN NN HD NN NI NI NN NN NN NG NI HD NG
**tal3b**	NI N* NI NI NN N* NI NK HD HD HD NG NI NN HD NN HD NG
**tal3c**	NN HD NI HD NN NG NG HD NN HD N* NN HD HD HD NG
**tal4b**	NS HG NI NG NS NN ND N* NG N* HN NN HD NS NI NN HD HD NG HD HG HD NG
**tal4a**	NI NG NI NG NS NN NG NN NG HD NN HD HD NG
**tal5a**	NN HD NS NG HD NN HD NI HD NN HD NN HD NN HD NN NN NN NN NN NN NN HD NG
**tal5b**	HH NN HD HD NG N* HD NN HD ND N* NG NS NS NN HD HD NG
**tal6**	NN HD NI NI NN HA NN NS NI NI HD HA HA HA HD HD HD HA HD N*
**tal7**	NN HD HD HH NN NG NS NG HD NI NG HD HH NG NG NG HA NG
**tal8**	NN NG HD NG HD HD NI NH HG NI NN NN HD NS NG NN HD NG NG N*
**tal9b**	NI NN HN NN NI NG HD NN HD HG HD HG HG HD HD NG
**tal9a**	NN NS NG NI HD HD NG NN HD NI NG N* N* NG HD HD NS NI NN NG NG
**tal10**	HD HG HD HG N* NN NG HD NN HD NG NG NN HD N* NG NG
**tal11b**	HD HD HD HD HD NG HD NN HD ND NG NG NN HG ND ND HD NG
**tal11a**	HD NN HD N* NS NN HG NI NI NS NI NG HD NN HD HD NG HG NG
**tal12**	NS HD NI N* NI HG NI NI N* NG HD NN NI NG HD NN NS NN NG NN

CFBP7331

**tal1'**	NN NG NI N* NS NG NN NN HG ND ND HD NG
**tal2**	NN HG HD NG HD QD NI NH HG NI NN NN HD NS NG NN HD NG NG NG NG
**tal3**	NN HD HD HH NN NG NS NG HD NI NG HD HH NG NG NG NG
**tal4**	NS HD NI NS NN HA NN NS NS NI HD HD HD HA HD NN HD NN HD N*
**tal5**	HD HD NN NN NG N* HD NI NG HD NG NG NS ND HA HD N* N* NN NI NN NN HD NG
**tal6a**	NI NG NI NG NN NG HH NG HD NN NN HG HD NN NS NN HD HD NG NA NN HD HD HD HD N*
**tal6b**	NN NG NI NN NG NN ND N* NG N* HN NN HD NN NI NN HD HD NG NG NG HD NG HD NG
**tal7c**	NN HD NI HD NN NG NG HD NN HD N* NN HD HD HD NG HD NN HD NS NI NG
**tal7b**	NN HD NI NI NG NN NN HD HH NI NI NN NN NN NG NI HD NG
**tal7a**	NI N* NI NI NN N* NI NK HD HD HD NG NI NN ND NN ND NG
**tal8a**	NI HG NI NG HN NG NN HD ND NG HD NI HG HG HD HH N* HH N* HD NN NG HD HD NN NN NG NN NI NV
**tal8b**	HD HD NN NN NG N* HD NI NG HD NG NS ND HA HD N* HH NG NI NN NN HD HD NG NN NG HD
**tal8c**	NI NG N* NN N* HD HG HD NN HD NN HG HG NS NG HD
**tal8d**	HD HG HD NI NN ND HD HD NN NN NI HD
**tal8e**	NN NN HD N* NS SN YG NI SN HD HD NG HD NS NN HD NG
**tal8f-**	NI HD HD N* HD H*
**tal9b**	NN NN HD NI NG HD HD HD NG NI HD HD NG
**tal9a**	NN NS NG NI HD HD NG NN HD NI NG N* N* NG HD HD NS NI NN NG N*
**tal10'**	NI SN YG NI SN HD HD NG HD NS #
**tal11b**	HD HD NI HD HD HG HD NN ND ND HG NG NN HG NG ND HD NG
**tal11a**	HD NN HD N* NS NN NG NI NI NG HY NG HD NN HD HD NG HG NG
**tal12**	NI N* NI HG NI NI N* NG HD NN NI NG HD NN NS NN NG NG

CFBP7341

**tal1**	NN NG NI N* NS NG NN NN HG ND ND HD NG
**tal2**	NN HG HD NG HD QD NI NH HG NI NN NN HD NS NG NN HD NG NG NG NG
**tal3**	NN HD HD HH NN NG NS NG HD NI NG HD HH NG NG NG NG
**tal4**	NS HD NI NS NN HA NN NS NS NI HD HD HD HA HD NN HD NN HD N*
**tal5'**	HD HD NN NN NG HA HD #
**tal6a**	NI NG NI NG NN NG HH NG HD NN NN HG HD NN NS NN HD HD NG NA NN HD HD HD HD N*
**tal6b**	NN NG NI NN NG NN ND N* NG N* HN NN HD NN NI NN HD HD NG NG NG HD NG
**tal7c**	NN HD NI HD NN NG NG HD NN HD N* NN HD HD HD NG HD NN HD NS NI NG
**tal7b**	NN HD NI NI NG NN NN HD HH NI NI NN NN NN NG NI HD NG
**tal7a**	NI N* NI NI NN N* NI NK HD HD HD NG NI NN ND NN ND NG
**tal8a**	NI HG NI NG HN NG NN HD ND NG HD NI HG HG HD HH N* HH N* HD NN NG HD HD NN NN NG NN NI NV
**tal8b**	HD HD NN NN NG N* HD NI NG HD NG NS ND HA HD N* HH NG NI NN NN HD HD NG NN NG HD
**tal8c**	NI NG N* NN N* HD HG HD NN HD NN HG HG NS NG HD
**tal8d**	HD HG HD NI NN ND HD HD NN NN NI HD
**tal8e**	NN NN HD N* NS SN YG NI SN HD HD NG HD NS NN HD NG
**tal8f-**	NI HD HD N* HD H*
**tal9b**	NN NN HD NI NG HD HD HD NG NI HD HD NG
**tal9a**	NN NS NG NI HD HD NG NN HD NI NG N* N* NG HD HD NS NI NN NG N*
**tal10'**	NI SN YG NI SN HD HD NG HD NS #
**tal11b**	HD HD NI HD HD HG HD NN ND ND HG NG NN HG NG ND HD NG
**tal11a**	HD NN HD N* NS NN NG NI NI NG HY NG HD NN HD HD NG HG NG
**tal12**	NI HD NI N* NI HG NI NI N* NG HD NN NI NG HD NN NS NN NG NG

CFBP7342

**tal1**	NI HD NI N* NI HG NI NI N* NG HD NN NI NG HD NN NS NN NG NG
**tal2a'**	HD NN HD N* NS NN NG NI NI NG HY NG HD NN HD HD NG HG NG
**tal2b**	HD HD NI HD HD HG HD NN ND ND NG NG NN HG HG ND HD NG
**tal3**	HD HG N* NN HG HD NN HD NN NG NN HD NN HD NG
**tal4a**	NN NS NG NI HD HD NG NN HD NI NG N* N* NG HD HD NS NI NN NG N*
**tal4b**	NI NN NI NI NG HD NG NN NI NN NI NN NG
**tal5**	NN HG HD NG HD HD NI NH HG NI NN NN HD NS NG NN HD NG NG NG NG
**tal6b**	NI HG NI NN NG NN ND N* NG N* HN NN HD HN NN NN HD HD NG NG NG HD NG
**tal6a**	NI HG NS NG NN NG HH NG HD NN NN HG HD NN NS NN HD HD NG NA NN HD HD HD HD N*
**tal7a**	HD HD NN NN NG N* HD NI NG HD NG NG NS ND HA HD N* HD NG NI NN NN HD HD NG HD
**tal7b**	HH NN HD HD NG N* HD NN HD ND N* NG NS NS NN HD HD NG
**tal8**	NN HD NS NS NN HA NN NS NS NI HD HD HD HA HD NN ND NN HD N*
**tal9**	NN HD HD HH NN NG NG NG HD NI NG HD HH NG NG NG NG
**tal10c**	NN HD NI HD NI NG NG HD NN HD N* NN HD HD NN HD HD NG
**tal10b**	NI N* NI NI NN N* NI NK HD HD HD NG NI NN ND N* HD NG
**tal10a**	NN HD NI NI NG NN NN HD HH NI NI NN NN NN NG NI HD NG
**tal11a**	NI HG NI NG HN NG NN HD ND NG HD NI HG NG HD HH N* HH N* HD NN NG HD HD NN NN NG NN NI NV
**tal11b**	HD HD NN NN NG N* HD NI NG HD NG NS ND HA HD N* HH NG NI NN NN HD HD NG HD
**tal11c**	NI NG N* NN N* HD HG HD NN HD NN HG HG NS NG HD
**tal11d**	HD HD NN NN NN NI NN NI NN NN HD HG NG
**tal11e**	NN HD NI N* NI NI NK HD HD HD NG HD NN HD HD
**tal11f**	NN NN HD N* NS SN YG NI SN HD HD NG HD NS NN HD NG
**tal11g'-**	NI HD N* HG NG HG HD HD N* HD H*
**tal12**	NN NG NS HD NI NG NN NN HG ND ND HG NG

L8

**tal1**	NS HD NI N* NI HG NI NI N* NG HD NN NI NG HD NN NS NN NG NN
**tal2a**	HD NN HD N* NS NN HG NI NI NS NI NG HD NN HD HD NG HG NG
**tal2b**	HD HD HD HD HD NG HD NN HD ND NG NG NN HG ND ND HD NG
**tal3**	HD HG HD HG N* NN NG HD NN HD NG NG NN HD N* NG NG
**tal4a**	NN NS NG NI HD HD NG NN HD NI NG N* N* NG HD HD NS NI NN NG NG
**tal4b**	NI NN NI HD NN NG HD NN HD HG HD HG HG HD HD NG
**tal5i-**	NI ND HG HG HG NG HG HG HD HD HD NN NN HD HG HH H* H* NN HD H*
**tal5h**	NN NN ND N* NS NS YG NI SN ND HD NG HD NS NN HD NG
**tal5g**	NN HD NI N* NI NI NK HD HD HD HG NI NN HD HD
**tal5f**	NI NN NI NI NI NI N* NI NG NN NN NN NG NS NG HD
**tal5e**	NI HG N* NI SN HD HD HD NN NI NK HD NN NN HD NG
**tal5d**	NI HG N* NN N* HD HG HD NN HD NN HG NG NS N* HD
**tal5c**	HD HD NN NN NG N* HD NI NG HD NG NS HD HA HD N* HD NN HD NN NN HD HD NG NN NG HD
**tal5b**	NI HG NI NG HN NG NN ND HD NG HD NI HG HG HD NN HD HH N* HD NN NN NG HD NN NN NG NN NI NG
**tal5a**	HD HD NC HD NC NG HD HH HD NI NG N* NS N* HD HD NS NI HG HD NG
**tal6a**	NN HD NI NI NG NN NN HD NN NI NI NN NN NN NG NI HD NG
**tal6b**	NI N* NI NI NN N* NI NK HD HD HD NG NI NN HD NN HD NG
**tal6c**	NN HD NI HD NN NG NG HD NN HD N* NN HD HD HD NG
**tal7**	NN HD NI NI NN HA NN NS NS NI HD HA HA HA HD HD HD HA HD N*
**tal8b**	HH NN HD HD NG N* HD NN HD ND N* NG NS NS NN HD HD NG
**tal8a**	NN HD NS NG HD NN HD NI HD NN HD NN HD NN HD NN NN NN NN NN NN NN HD NG
**tal9a**	NI NG NI NG NN NG NN NG HD NN NN HG HD NN NS NN HD HD NG NA NN HD HD HD HD N*
**tal9b**	NS HG NS NG NS NN NG NN NG HD NN HD HD NG
**tal9c**	NS HG NI NG NS NN ND N* NG N* HN NN HD NS NS NN NG NN NG HD NN HD HD NG
**tal9d**	NS HG NI NG NS NN ND N* NG N* HN NN HD NS NI NN HD HD NG NG HG HD NG
**tal10**	NN HD HD HH NN NG NS NG HD NI NG HD HH NG NG NG HA NG
**tal11**	NN NG HD NG HD HD NI NH HG NI NN NN HD NS NG NN HD NG NG N*
**tal12a**	NI NN NI NI NG HD NG NN NI NN NI NN HD
**tal12b**	HD HD HD HD HD NG HD NN HD NG HG NN HD N* NG NG

RS105

**tal1**	NS HD NI N* NI HG NI NI N* NG HD NN NI NG HD NN NS NN NG NN
**tal2a**	HD NN HD N* NS NN HG NI NI NS NI NG HD NN HD HD NG HG NG
**tal2b**	HD HD HD HD HD NG HD NN HD ND NG NG NN HG ND ND HD NG
**tal3**	HD HG HD HG N* NN NG HD NN HD NG NG NN HD N* NG NG
**tal4a**	NN NS NG NI HD HD NG NN HD NI NG N* N* NG HD HD NS NI NN NG NG
**tal4b**	NI NN NI HD NN NG HD NN HD HG HD HG HG HD HD NG
**tal5e-**	NI ND HG HG HG HG NG HG HG HD HD HD HD NN NN HD HG HH H* H* NN HD H*
**tal5d**	NN NN ND N* NS NS YG NI SN ND HD NG HD NS NN HD NG
**tal5c**	NN HD NI N* NI NI NK HD HD HD HG NI NN HD HD
**tal5b**	NI HG NI NG HN NG NN ND HD NG HD NI HG HG HD NN HD HH N* HD NN NN NG HD NN NN NG NN NI NG
**tal5a**	HD HD NC HD NC NG HD HH HD NI NG N* NS N* HD HD NS NI HG HD NG
**tal6a**	NN HD NI NI NG NN NN HD NN NI NI NN NN NN NG NI HD NG
**tal6b**	NI N* NI NI NN N* NI NK HD HD HD NG NI NN HD NN HD NG
**tal6c**	NN HD NI HD NN NG NG HD NN HD N* NN HD HD HD NG
**tal7**	NN HD NI NI NN HA NN NS NS NI HD HA HA HA HD HD HD HA HD N*
**tal8b**	HH NN HD HD NG N* HD NN HD ND N* NG NS NS NN HD HD NG
**tal8a**	NN HD NS NG HD NN HD NI HD NN HD NN HD NN HD NN NN NN NN NN NN NN HD NG
**tal9a**	NI NG NI NG NN NG NN NG HD NN NN HG HD NN NS NN HD HD NG NA NN HD HD HD HD N*
**tal9b**	NS HG NI NG NS NN NG NN NG HD NN HD HD NG
**tal9c**	NS HG NI NG NS NN ND N* NG N* HN NN HD NS NI NN HD HD NG NG HG HD NG
**tal10'**	NN HD HD HH NN NG NS NG HD NI NG HD HH NG NG NG HA NG
**tal11**	NN NG HD NG HD HD NI NH HG NI NN NN HD NS NG NN HD NG NG N*
**tal12a**	NI NN NI NI NG HD NG NN NI NN NI NN HD
**tal12b**	HD HD HD HD HD NG HD NN HD NG HG NN HD N* NG NG
