# Supplementary material for: TAL effectors and activation of predicted host targets distinguish Asian from African strains of the rice pathogen Xanthomonas oryzae pv. oryzicola while strict conservation suggests universal importance of five TAL effectors
Source: Front Plant Sci. 2015 Jul 21;6:536. doi: 10.3389/fpls.2015.00536 (PMC4508525; doi:10.3389/fpls.2015.00536)
Supplement: Supplementary file 2 [file DataSheet2.PDF]

Supplementary Material

TAL effector content and host transcriptional response across diverse strains of the rice bacterial leaf streak pathogen *Xanthomonas oryzae* pv. oryzicola

Katherine Wilkins[1,2], Nicholas J. Booher[1,2], Li Wang[1], Adam J. Bogdanove[1*]

[1]Plant Pathology and Plant-Microbe Biology Section, School of Integrative Plant Science, Cornell University, Ithaca, NY, USA
[2]Graduate Field in Computational Biology, Cornell University, Ithaca, NY, USA

*** Correspondence:** Adam J. Bogdanove, Plant Pathology and Plant-Microbe Biology Section, School of Integrative Plant Science, Cornell University, 334 Plant Science Building, Ithaca, NY, 14853, USA.
ajb7@cornell.edu

1. Supplementary Figures and Tables

1.1. Supplementary Tables

Supplementary Table S1. Machine learning classifier performance with manual versus automated annotation.

Annotation method	Accuracy	Precision	Recall	F-measure	MCC	ROC area
**Manual**	.89	.88	.92	.90	.77	.88
**Automated**	.79	.77	.87	.82	.47	.85

1.2. Supplementary Figures

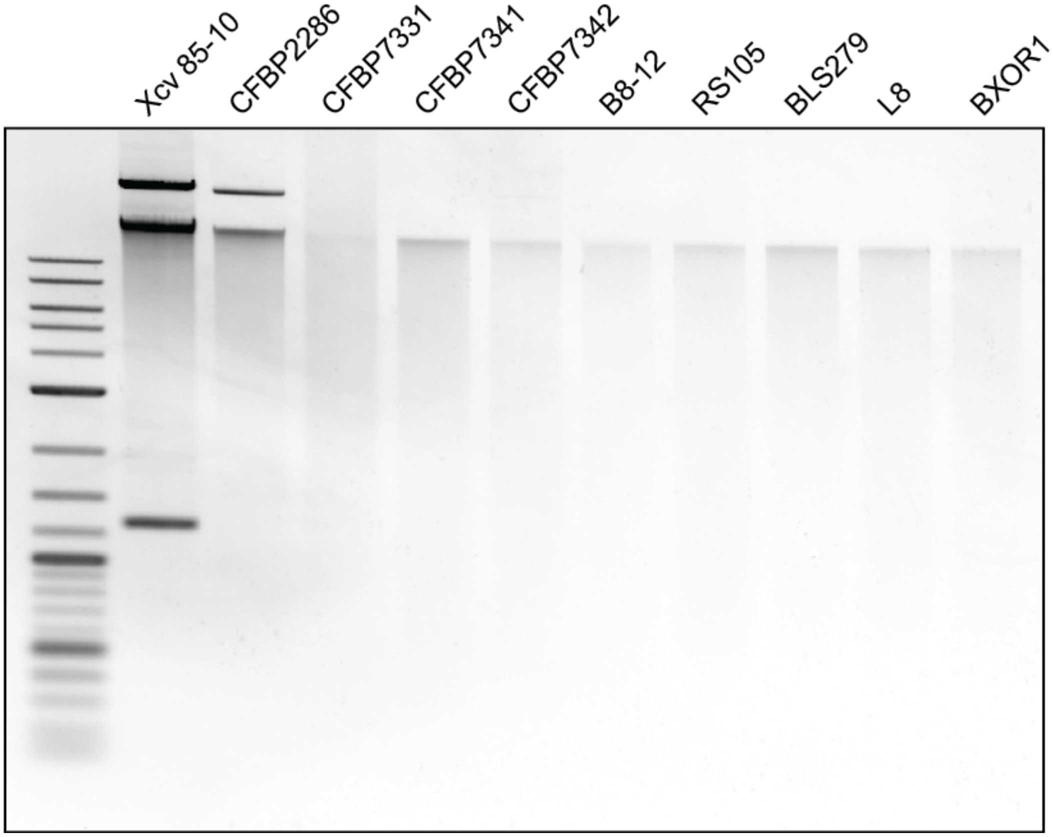

Supplementary Figure S1. Plasmid profiles of the 10 Xoc strains. Strains were cultured and DNA was prepared and subjected to agarose gel electrophoresis as described (Chakrabarty et al., 2010), except that the E.Z.N.A. Plasmid DNA Mini Kit I (Omega Bio-Tek, Norcross, GA) was used for DNA isolation. *X. campestris* pv. vesicatoria strain 85-10 (Thieme et al., 2005) was included as a positive control. The leftmost lane contains 2-Log Ladder (New England Biolabs, Ipswich, MA).

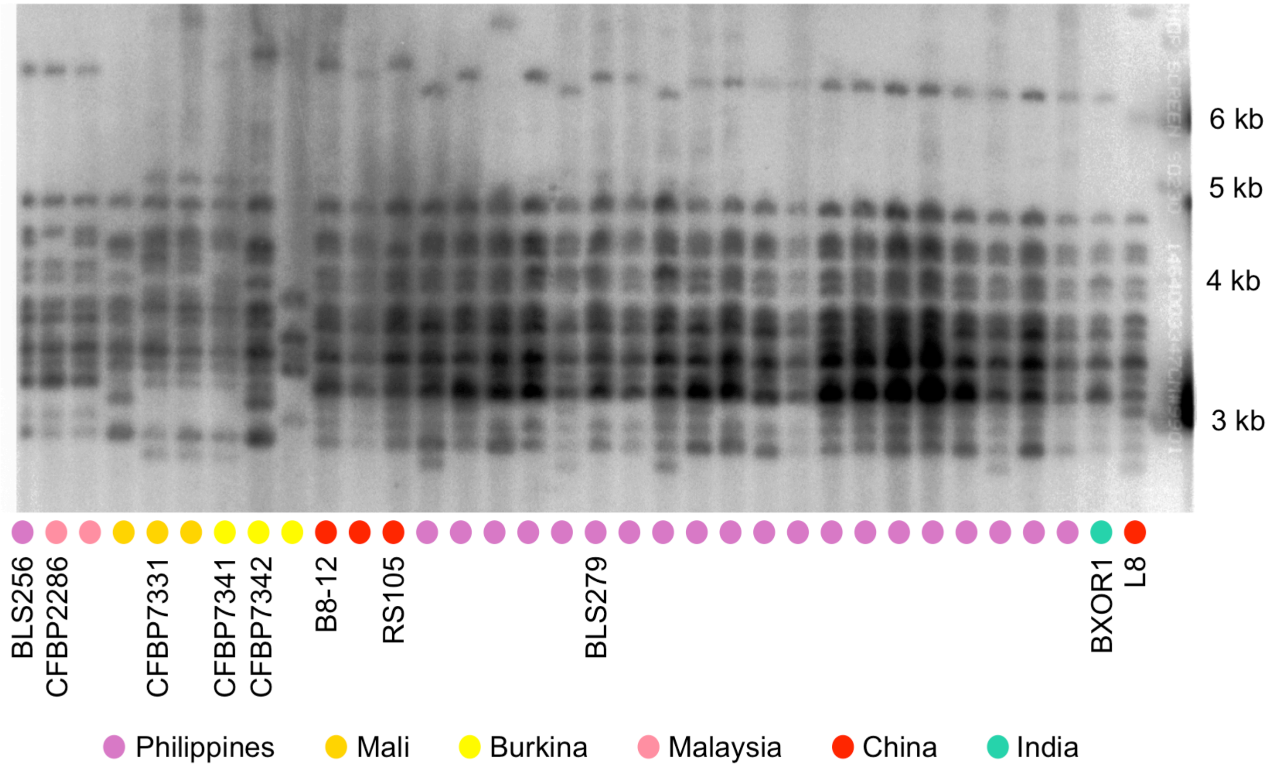

Supplementary Figure S2. Southern blot of *Bam*HI-digested genomic DNAs of geographically diverse Xoc strains probed with a DNA fragment encoding 6 TAL effector repeats. The strains examined in this study are labeled. Colored dots indicate the country of isolation, as indicated at bottom.

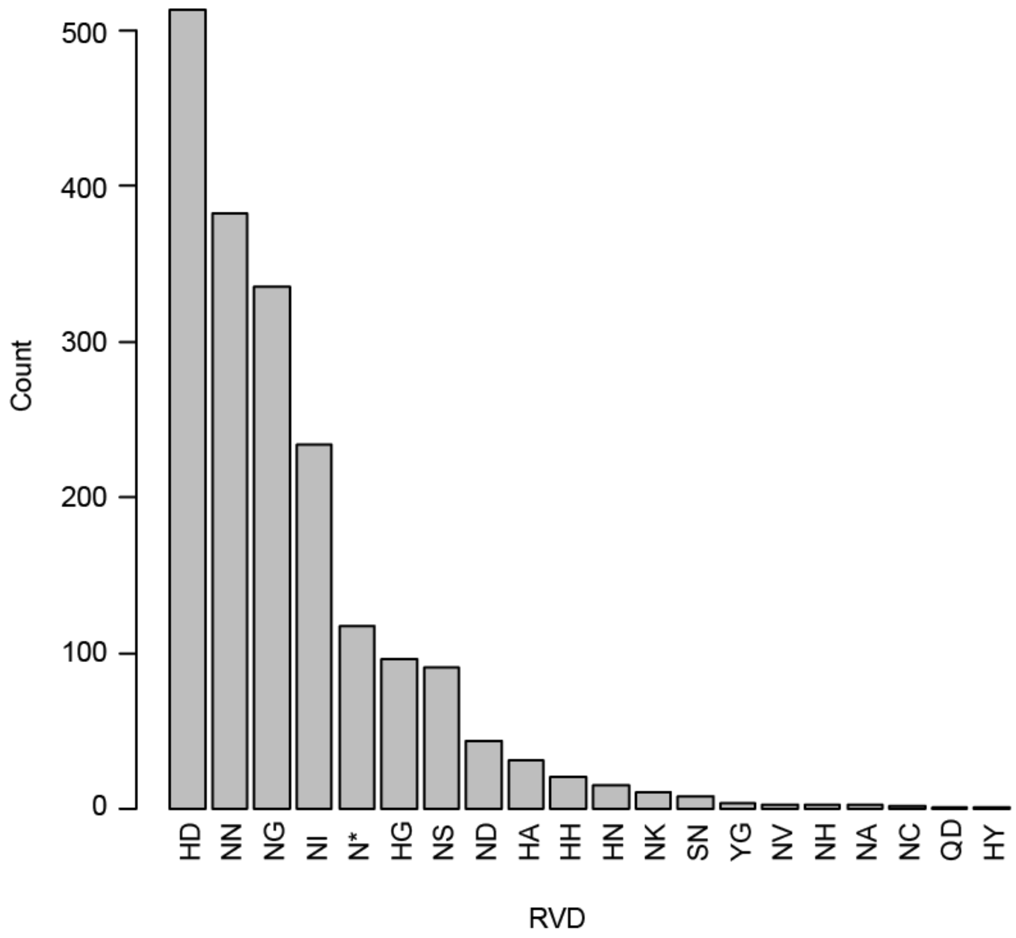

Supplementary Figure S3. RVDs present in the intact TAL effectors of all 10 Xoc strains.

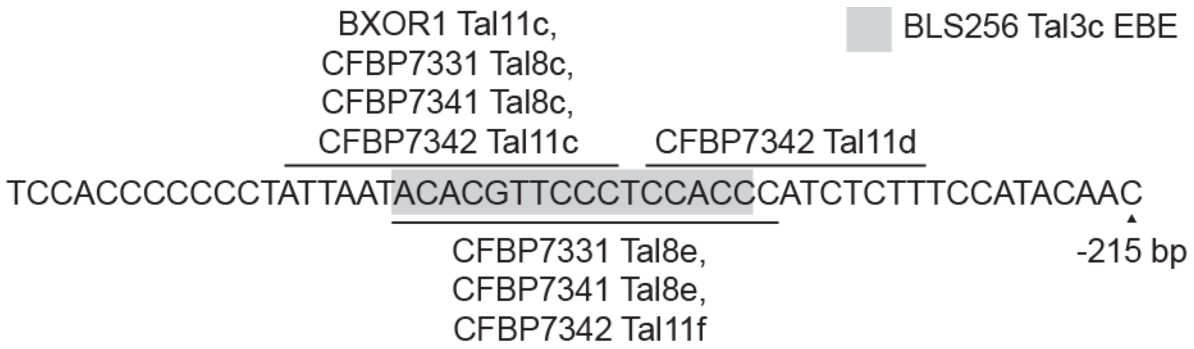

Supplementary Figure S4. Predicted EBEs for distinct TAL effectors from the three African Xoc strains and the Indian strain BXOR1 in the promoter of *Os03g07540*, a target of BLS256 Tal3c. The BLS256 Tal3c EBE is shown in gray and the others are denoted by labeled black lines. The sequence is shown 5’ to 3’ and the coordinate shows the distance from the translational start site. The transcriptional start site is unannotated.

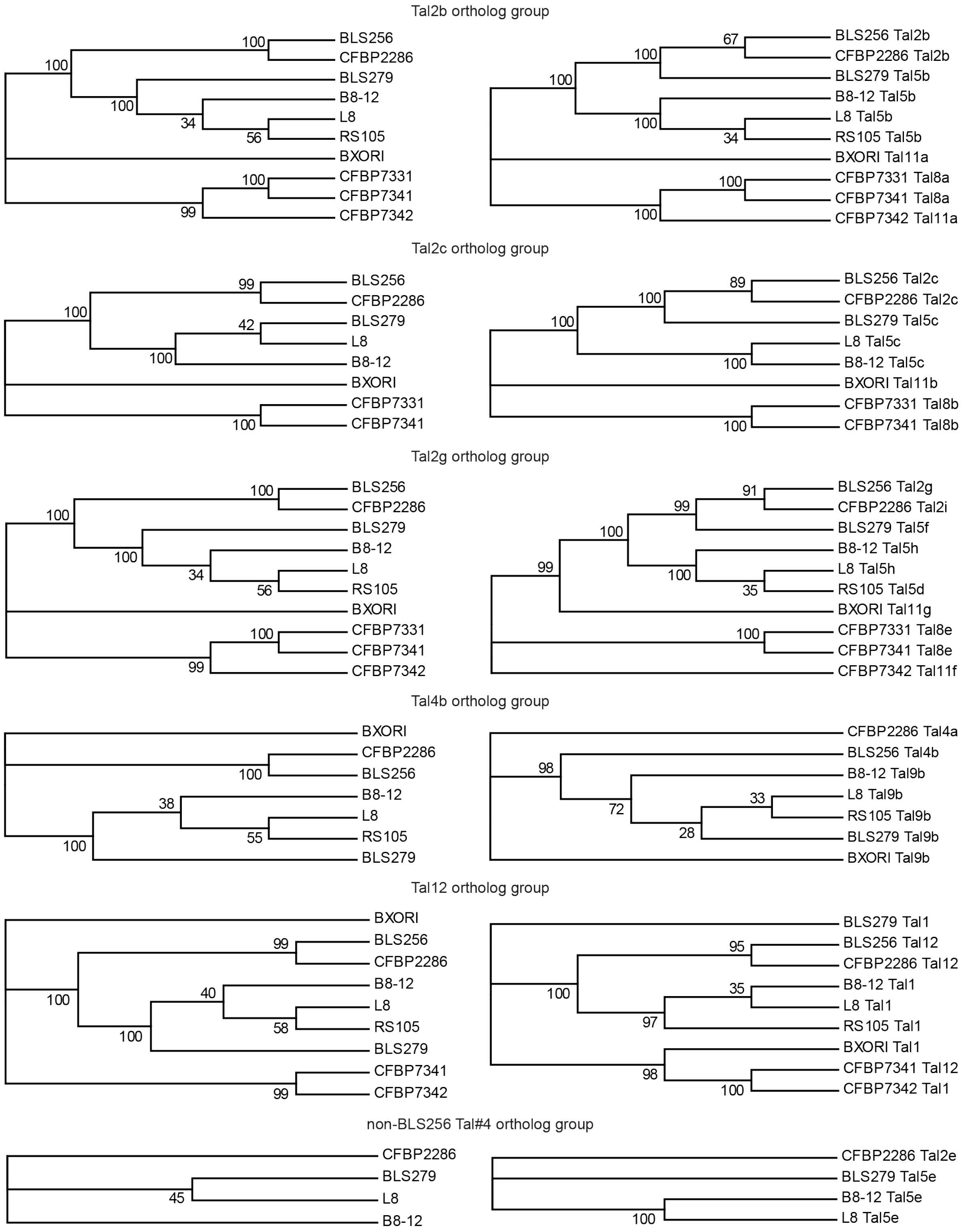

Supplementary Figure S5. Phylogenetic relationships based on 31 housekeeping genes vs. TAL effectors for the TAL effector ortholog groups for which the two are significantly different. Maximum likelihood trees based on the housekeeping genes (left) and the TAL effector genes (right) are shown, with bootstrap values from 1,000 replicates given at the nodes (see Methods).

References

Chakrabarty, P.K., Chavhan, R.L., Ghosh, A., and Gabriel, D.W. (2010). Rapid and efficient protocols for throughput extraction of high quality plasmid DNA from strains of *Xanthomonas axonopodis* pv *malvacearum* and *Escherichia coli*. *Journal of Plant Biochemistry and Biotechnology* 19**,** 99-102. doi: 10.1007/bf03323444.

Thieme, F., Koebnik, R., Bekel, T., Berger, C., Boch, J., Büttner, D., Caldana, C., Gaigalat, L., Goesmann, A., Kay, S., Kirchner, O., Lanz, C., Linke, B., Mchardy, A.C., Meyer, F., Mittenhuber, G., Nies, D.H., Niesbach-Klösgen, U., Patschkowski, T., Rückert, C., Rupp, O., Schneiker, S., Schuster, S.C., Vorhölter, F.-J., Weber, E., Pühler, A., Bonas, U., Bartels, D., and Kaiser, O. (2005). Insights into genome plasticity and pathogenicity of the plant pathogenic bacterium *Xanthomonas campestris* pv. *vesicatoria* revealed by the complete genome sequence. *J Bacteriol* 187**,** 7254-7266. doi: 10.1128/jb.187.21.7254-7266.2005.
